# The Dynamics and Prognostic Potential of DNA Methylation Changes at Stem Cell Gene Loci in Women's Cancer

**DOI:** 10.1371/journal.pgen.1002517

**Published:** 2012-02-09

**Authors:** Joanna Zhuang, Allison Jones, Shih-Han Lee, Esther Ng, Heidi Fiegl, Michal Zikan, David Cibula, Alexandra Sargent, Helga B. Salvesen, Ian J. Jacobs, Henry C. Kitchener, Andrew E. Teschendorff, Martin Widschwendter

**Affiliations:** 1 Department of Women's Cancer, University College London Elizabeth Garrett Anderson Institute for Women's Health, London, United Kingdom; 2 Statistical Genomics Group, University College London Cancer Institute, London, United Kingdom; 3 Department of Gynaecology and Obstetrics, Innsbruck Medical University, Innsbruck, Austria; 4 Oncogynecologic Center, Department of Obstetrics and Gynaecology, Charles University Prague–First Faculty of Medicine and General Faculty Hospital, Prague, Czech Republic; 5 School of Cancer and Imaging Science, Manchester Academic Health Science Centre, Central Manchester University Hospitals NHS Foundation Trust, Manchester, United Kingdom; 6 Department of Obstetrics and Gynaecology, Haukeland University Hospital, Bergen, Norway; 7 Department of Clinical Medicine, University of Bergen, Bergen, Norway; HudsonAlpha Institute for Biotechnology, United States of America

## Abstract

Aberrant DNA methylation is an important cancer hallmark, yet the dynamics of DNA methylation changes in human carcinogenesis remain largely unexplored. Moreover, the role of DNA methylation for prediction of clinical outcome is still uncertain and confined to specific cancers. Here we perform the most comprehensive study of DNA methylation changes throughout human carcinogenesis, analysing 27,578 CpGs in each of 1,475 samples, ranging from normal cells in advance of non-invasive neoplastic transformation to non-invasive and invasive cancers and metastatic tissue. We demonstrate that hypermethylation at stem cell PolyComb Group Target genes (PCGTs) occurs in cytologically normal cells three years in advance of the first morphological neoplastic changes, while hypomethylation occurs preferentially at CpGs which are heavily Methylated in Embryonic Stem Cells (MESCs) and increases significantly with cancer invasion in both the epithelial and stromal tumour compartments. In contrast to PCGT hypermethylation, MESC hypomethylation progresses significantly from primary to metastatic cancer and defines a poor prognostic signature in four different gynaecological cancers. Finally, we associate expression of TET enzymes, which are involved in active DNA demethylation, to MESC hypomethylation in cancer. These findings have major implications for cancer and embryonic stem cell biology and establish the importance of systemic DNA hypomethylation for predicting prognosis in a wide range of different cancers.

## Author Summary

DNA methylation is an important chemical modification of DNA that can affect and regulate the activity of genes in human tissue. Abnormal DNA methylation and its subsequent effects on gene activity are a hallmark of cancer, yet when precisely these DNA methylation changes occur and how they contribute to the development of cancer remains largely unexplored. In this work we measure the methylation state of DNA at over 14,000 genes in over 1,475 samples, including normal and benign cells, invasive cancers, and metastatic cancer tissue. Using cervical cancer as a model, we show that gain of abnormal methylation at genes typically un-methylated in stem cells can be detected up to 3 years in advance of the appearance of pre-cancerous cells, while those genes typically methylated in stem cells lose this methylation progressively throughout cancer development. Furthermore, we discover that this process of methylation loss during cancer progression is a marker of poor disease outcome common to all four major women-specific cancers: breast, ovarian, endometrial, and cervical cancers. Finally we demonstrate the relationship between loss of methylation and cancer-specific over-production of a specific protein known to play an active role in removing methylation from DNA. Taken together these findings highlight the complex nature of DNA methylation dynamics in cancer development as well as their potential exploitation for clinical gain.

## Introduction

Aberrant DNA methylation is one of the most important cancer hallmarks [1], yet its precise role in carcinogenesis and clinical prognosis remains ill-defined [2]. Indeed, the dynamical changes in DNA methylation that happen during carcinogenesis, in particular those prior to morphological changes, have not yet been explored in detail. Moreover, no study has so far reported a DNA methylation signature capable of predicting prognosis across multiple human cancers, unlike gene expression and DNA copy number where such prognostic signatures have been described [3], [4].

Both hyper and hypomethylation are commonly observed in cancer [1]. In contrast to hypomethylation, which seems to target large inter-genic satellite repeat regions, hypermethylation appears to happen locally, preferentially targeting the promoters of genes. Several studies have reported that a statistically high fraction of these promoters map to stem cell PolyComb Group Target genes (PCGTs) [5], [6], many of which encode transcription factors needed for differentiation, and which are normally suppressed in embryonic stem cells through a reversible mechanism mediated by the Polycomb Repressive Complex (PRC2) [7]. This preferential hypermethylation at PCGTs in cancer supports the view that the reversible gene repression of PCGTs in stem cells may be replaced by permanent silencing in cancer, potentially impairing the differentiation capacity of cells [1], [5], [6]. Although there is no causal functional data linking PCGT methylation to carcinogenesis yet, there is accumulating evidence that factors which lead to cancer, for instance age or oxidative damage, are causally involved in DNA methylation at PCGTs [8]–[11].

Another feature of the epigenetic landscape characterising human embryonic stem cells (hESC) was described by Lister et al [12]. Specifically, using single-base-resolution DNA methylation maps, they demonstrated that a substantial fraction of CpGs is heavily (>80%) Methylated in human Embryonic Stem Cells (MESC) (see Materials and Methods for the precise definition of MESC CpGs and Table S1 for the complete list of MESC CpGs on the 27 k array). However, it is unknown at present what role MESCs may play throughout carcinogenesis. Thus, which epigenetic stem cell features are retained or changed in human cancer and even more importantly at which stage during human carcinogenesis these epigenetic changes occur, is still unclear.

Motivated by these outstanding questions, we decided to (i) explore the dynamics of epigenetic changes at stem cell loci (PCGTs and MESCs) throughout all stages of human carcinogenesis and (ii) to investigate their potential role in predicting poor prognosis.

To address our first aim, we used as a model the uterine cervix, since screening programs in place allow easy access to this organ, and cervical carcinogenesis is also one of the few scenarios in humans where DNA methylation changes in the actual cell of origin and occurring throughout disease progression can be analyzed. Specifically, we measured DNA methylation at over 27,000 CpGs in cervical cells and at three different stages: (a) three years before onset of dysplastic changes, (b) at the stage of non-invasive dysplasia, and (c) at the stage of invasive cervical cancer. To address our second aim we analysed DNA methylation data from 5 independent cohorts encompassing a total of 1,026 tumour samples in 4 different gynaecological cancers. In total, we analysed DNA methylation data from 10 independent studies, encompassing normal and cancer tissue from 5 different tissue types, including metastases (Table 1).

**Table 1:** Data sets used in this study.

Data set	Sample size	Normal	Cancer	Cell type	Age range (yr)	Reference
BDy	152	152	0	LBC	19–55	GSE30760
Dy	48	30	18	LBC	26–43	GSE20080
Cvx CA	63	15	48	tissue	24–91	GSE30760
BC	60	37	23	tissue	19–75	GSE32393
BC (JHU)	118	15	103	tissue	20–96	GSE31979
EC	87	64	23	tissue	32–90	Salvesen,H.B. et al.
EC (Meta)	17	0	17	tissue	43–93	GSE33422
OvC	177	0	177	tissue	24–88	Teschendorff, A.E. et al.
OvC (TCGA)	378	0	378	tissue	34–89	TCGA
ColC	154	29	125	tissue	NA	GSE25062
LC	151	24	127	tissue	NA	TCGA
LC (Fibro)	10	5	5	fibroblast	58–77	GSE22874
*BRCA1*	60	60	0	WBC	50–80	GSE32396

Cervical cells collected three years before half of the patients developed dysplasia (BDy), normal versus dysplastic cervical cells (Dy), normal cervical tissue and invasive cervical cancer (Cvx CA), non-neoplastic breast tissue and breast cancer (BC), normal endometrium and endometrial cancer (EC) and metastatic endometrial cancer (EC (Meta)), ovarian cancer tissue (OvC; The Cancer Genome Atlas, TCGA), colon cancer tissue (ColC), lung cancer tissue (LC) and normal and cancer associated lung fibroblasts (LC Fibro), and white blood cell (WBC) samples from *BRCA1* mutation carriers and controls (*BRCA1*). The number of samples, their distribution in terms of normal and cancer, cell-type, age-range (years) and reference to data access is given. GSE numbers are GEO accessions. NA, not available.

Using these data we here report four major novel aspects of cancer epigenetics: (i) Hypermethylation at PCGT stem cell loci occurs up to three years before the first signs of morphological transformation, (ii) hypomethylation at MESC stem cell loci is a hallmark of cancer invasion, affecting both epithelial and stromal compartments, and increases further in metastases, (iii) hypomethylation instability at MESCs defines a stem cell DNA methylation signature that predicts poor prognosis in multiple human cancers independently of standard prognostic factors, and (iv) expression of TET enzymes [13]–[17] is strongly associated with MESC hypomethylation.

## Results

All methylation data in this study were generated with the Illumina Infinium Human Methylation27 beadchip array (Materials and Methods), which assesses the DNA methylation status of 27,578 CpG sites located in the promoter regions of 14,495 genes as described previously [18]. Among these CpGs, 3,465 map to PCGTs, whilst 5,943 map to MESC CpGs (Materials and Methods, Table S1 and Table S2). We also made a distinction between CpGs located within Partially Methylated Domains (PMDs) (a total of 4,750 CpGs on the array mapped to PMDs), and those that are not (termed non-PMDs). PMDs demonstrate reduced methylation levels in more differentiated embryonic tissue compared to embryonic stem cells, and consist of focally hypermethylated elements (corresponding overwhelmingly to CpG islands), concentrated within regions of long-range hypomethylation [12]. PMDs were recently described also in cancer [19]. For precise definitions see Text S1.

To investigate the dynamics of DNA methylation in human carcinogenesis we designed a study with samples from three different phases reflecting cervical carcinogenesis: (1) ‘Before Dysplasia (BDy)’: normal cervical epithelial cells collected within the ARTISTIC trial [20], [21] (n = 152) of which 75 developed a cervical intraepithelial neoplasia grade 2 or 3 (CIN2/3) *after* three years (cases), whereas the other 77 remained normal (controls). These samples were matched for age and HPV status. (2) ‘Dysplasia (Dy)’: age-matched non-invasive dysplastic epithelial cells (CIN2/3) (n = 18, all HPV+) and normal cervical epithelial cells (n = 30, 19 HPV− and 11 HPV+) collected within screening programs [22], and (3) ‘Invasive Cancer (CA)’: invasive cervical cancer tissue (n = 48) and normal cervical tissue (n = 15) collected within a clinical setting. Further details of the samples are described in Text S1 (see also Table 1).

**Figure 1:**
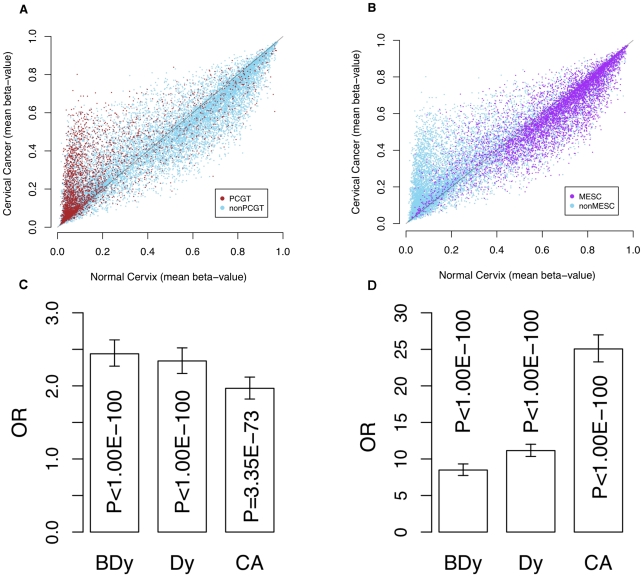
Methylation profile of PCGTs and MESCs in cervix data. Scatter plots of mean β-values in normal cervical tissue (x-axes) vs. cervical cancer tissue (y-axes) of (A) all CpGs with PCGTs highlighted in brown and (B) all CpGs with MESCs highlighted in purple. (C) is the bar chart indicating the enrichment odds ratios (OR) and P-values (Fisher-test), testing for enrichment of PCGTs among CpGs unmethylated in normal cervix (mean β-value<0.2) and with a higher mean β-value in (i) normal samples which develop dysplasia (BDy), (ii) non-invasive dysplastic samples (Dy), and (iii) invasive cervical cancer (CA); (D) the bar chart indicating the enrichment of MESCs among those CpGs methylated in the normal cervix (mean β-value>0.4) and with a lower mean β-value in tissue representing the three stages of cervical carcinogenesis (BDy, Dy, and CA).

**Figure 2:**
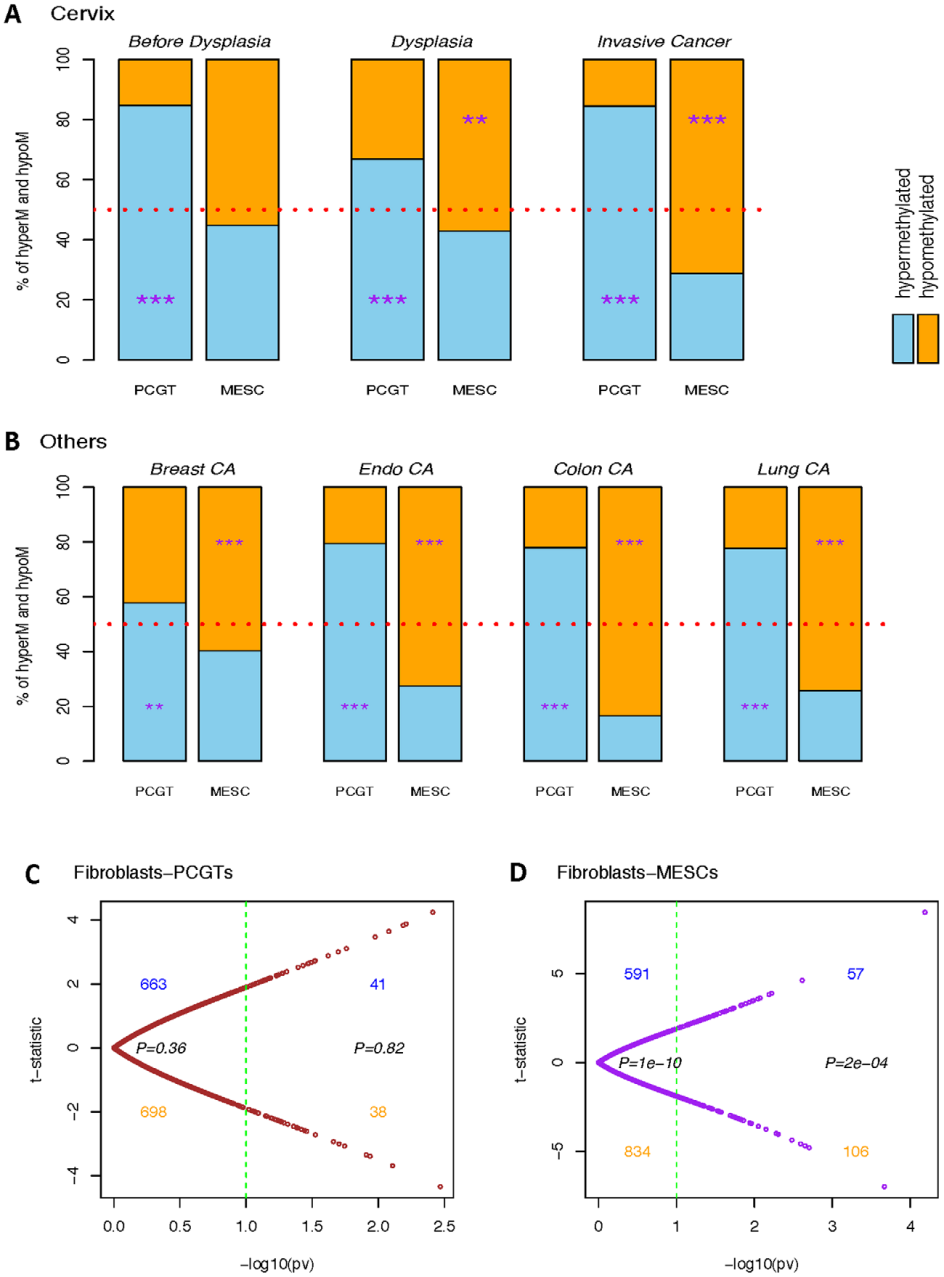
Differential dynamics of hypermethylated and hypomethylated PCGTs and MESCs. Bar charts representing percentages of significantly hypermethylated (blue) and hypomethylated (orange) PCGT and MESC CpGs in (A) each stage of cervical carcinogenesis: Cervix ‘Before Dysplasia’, ‘Dysplasia’, and ‘Invasive Cancer’, all relative to normal cervical cells or tissue; and in (B) ‘Breast CA’, ‘Endo CA’, ‘Colon CA’, and ‘Lung CA’, all relative to their respective normal controls. The significance of the binomial test assessing skew of hypermethylated versus hypomethylated is indicated by ‘*’, ‘**’, and ‘***’ for P-value<0.05, 0.01, and 0.001 respectively. (C) and (D) are the scatterplots of the age-adjusted linear regression t-statistics against their corresponding −log10(P-values) testing the association with the normal and lung cancer fibroblasts on the colon-PMD PCGTs and colon-PMD MESCs respectively.

As expected, PCGTs were highly enriched among CpGs hypermethylated in invasive cervical cancer (Figure 1A and 1C). In contrast, CpGs that become hypomethylated in invasive cervical cancer are to a large extent MESCs (Figure 1B and 1D). Most importantly, PCGTs were hypermethylated three years prior to any cytological changes (Figure 1C, OR = 2.44; 95%CI = 2.27–2.63; p<10^−100^), especially for those PCGT CpGs located within PMDs (OR = 4.81; 95%CI = 4.19–5.52; p<10^−100^). We verified that PCGT enrichment was also independent of HPV status (P<0.005 for HPV+ and HPV−). Notably, the frequency of hypermethylation remained fairly constant throughout the phases from non-invasive dysplasia to invasive cancer (Figure 2A and Figures S1, S2, S3, S4).

In contrast to PCGT methylation, MESC hypomethylation appears as a progressive process towards invasive cancer: whereas we observed a substantial enrichment of MESCs in the normal samples three years prior to the dysplastic changes (OR = 5.69 and 9.55 for PMD and nonPMD respectively), non-invasive dysplastic samples had an increased MESC enrichment in hypomethylated CpGs (OR = 7.62 and 12.30 for PMD and nonPMD, respectively) and eventually MESC CpGs contributed most significantly to hypomethylated CpGs in invasive cancer (OR = 18.84 and 26.85 for PMD and nonPMD respectively; Figure 1D, Figure 2A, and Figures S1, S2, S3, S4). In order to check that these enrichments are not just a consequence of the baseline methylation levels (i.e. the levels in normal tissue), we estimated the enrichment relative to other CpGs with specific baseline methylation levels (CpGs with mean β-values in normal cervical tissue samples of <0.2 and >0.4). This confirmed that the observed PCGT and MESC enrichment was independent of the initial methylation levels in normal tissue, and that this was particularly true for PCGT/MESC CpGs within PMDs (Figure S5). Thus, MESC CpGs that showed reduced methylation levels (<80%) in normal tissue compared to their levels in hESCs (>80%) were still more likely to exhibit further hypomethylation in dysplasia and cancer than a control set of CpGs with similar methylation levels in normal tissue (Figure S5).

To test if PCGT and MESC methylation changes are also present in cells which are not immediately involved in carcinogenesis we studied white blood cell DNA from women who carry *BRCA1* mutations and who are therefore at an 80% lifetime risk of developing breast and/or ovarian cancer. Whereas MESC methylation was not altered, we observed that PCGTs were highly enriched among CpGs hypermethylated in blood cells from *BRCA1* mutation carriers (Figures S6 and S7).

Next, we asked if the progressive hypomethylation of MESCs towards invasive cancer is a generic feature of tumour biology. We analysed DNA methylation profiles of breast, endometrial, colorectal and lung cancer (Text S1; Figure 2B and Figures S1, S2, S6, S7), and in all cancer types we observed a significant loss of methylation at MESC CpGs, concurrent with the expected hypermethylation of PCGT CpGs.

As demonstrated in Figure 2A and 2B, PCGT methylation enrichment exists prior to and at the stage of non-invasive dysplasia when analyzing only epithelial cells without stroma and remains constant when studying invasive cancer tissue which contains some stromal components. In contrast, MESC enrichment doubles in the hypomethylated fraction when comparing invasive cancer to non-invasive dysplastic cells. This pronounced enrichment could be contributed by MESC hypomethylation in the cancer-associated stromal component. To test this, we analyzed those PCGTs and MESCs that are enriched in the hyper- and hypomethylated fractions in lung cancer and asked if these CpGs are also enriched in lung cancer associated fibroblasts compared to normal lung fibroblasts [23]. Interestingly, while there was no enrichment of PCGTs (Figure 2C), there was a clear enrichment of lung cancer MESCs among PMD CpGs that are hypomethylated in lung cancer fibroblasts (Figure 2D). This further supports the view that MESC hypomethylation is an important characteristic of cancer invasion, and that it may therefore be a molecular determinant of clinical outcome.

**Figure 3:**
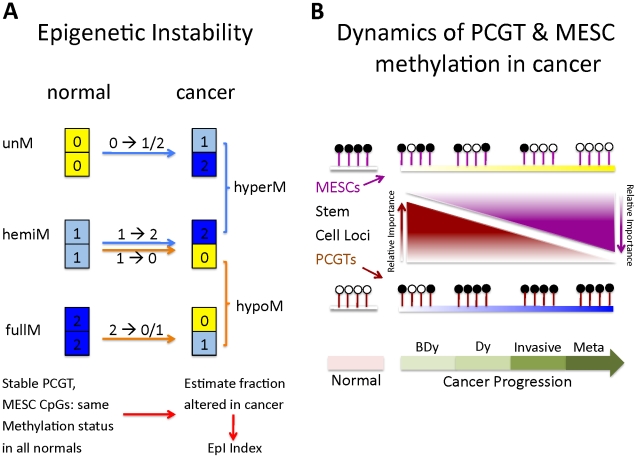
Definition of epigenetic instability indices and dynamics of PCGT and MESC methylation in cancer. (A) Definition of epigenetic instability indices. Shown are the six possible DNA methylation changes between normal and cancer tissue. Thresholds used to define unmethylated (yellow), hemimethylated (skyblue) and fully methylated (blue) CpGs are described in Materials and Methods. Stable MESC (or PCGT) CpGs are defined by MESC (or PCGT) CpGs, which have the same methylation state in all normal samples. The Epigenetic Instability Index (EpI) is then defined as the fraction of stable CpGs altered in cancer. We defined 4 separate indices to describe the transitions: 0→1/2, 1→2, 1→0, 2→1/0. The index describing alterations from a fully methylated to either a hemi or unmethylated state is called the Demethylation instability index (DeMI). (B) Dynamics of PCGT and MESC DNA methylation in cancer. Diagram illustrates the differential dynamics of PCGT and MESC CpG DNA methylation in cancer. Most PCGTs start out unmethylated (white lolly-pops) in normal cells but acquire DNA methylation (black lolly-pops) in normal cells 3 years before the emergence of dysplasia (BDy). PCGT hypermethylation increases further with Dysplasia (Dy) and cancer, but is not a strong determinant of invasion or poor outcome (metastasis). In contrast, most MESCs start out either fully or hemi methylated in normal cells, and gradually lose methylation during the progressive stages of cancer, with hypomethylation at MESCs a key determinant of metastases and poor outcome.

**Figure 4:**
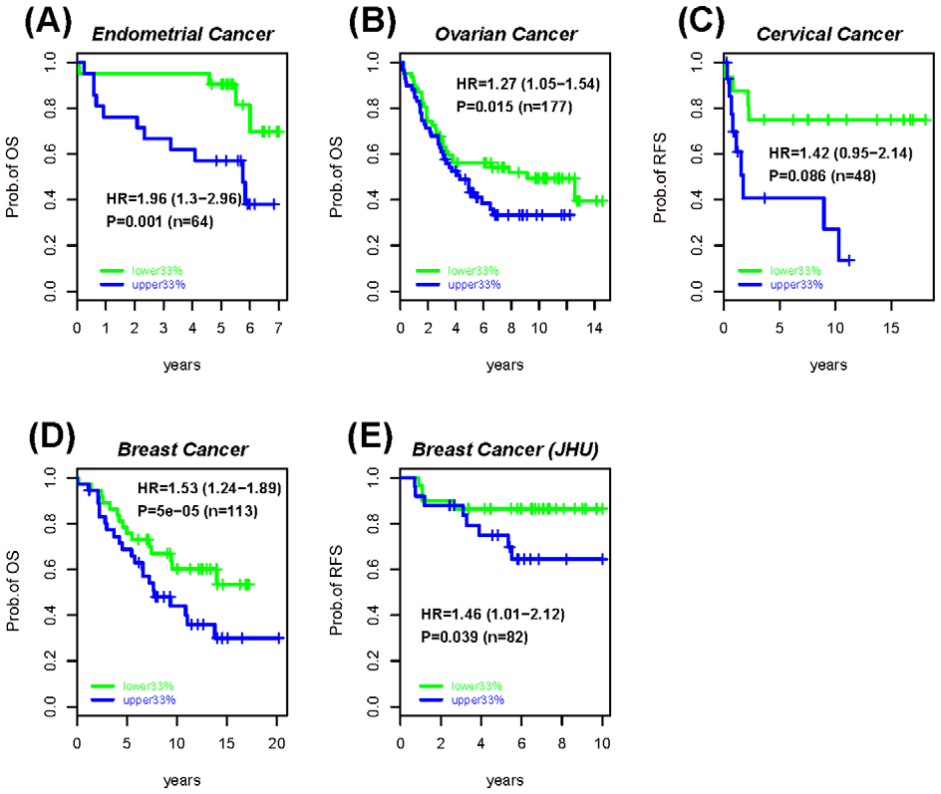
Survival analysis of the MESCs demethylation instability index in various cancers. Kaplan-Meier survival curves between the upper (blue) and lower (green) tertiles of the demethylation instability index (DeMI) at MESCs in (A) Endometrial cancer, (B) Ovarian cancer, (C) Cervical cancer, and (D–E) two Breast Cancer cohorts. The hazard ratio (HR), 95% confidence interval (CI) and P-values are from the multivariate Cox regression model, with “n” denoting the number of samples in cohort. Clinical endpoint used is indicated on the y-axis (OS = overall survival, RFS = relapse free survival).

Molecular signatures, and in particular gene expression signatures, involving stem cell genes have been associated with poor prognosis in several cancers [24], [25]. Therefore, given the fundamental role of PCGT and MESC CpGs in the dynamics of DNA methylation in human cancer, as just described, it is natural to ask if DNA methylation changes at these stem cell loci can predict clinical outcome. In particular, we posited that epigenetic instability, as measured by DNA methylation changes from a normal reference, might indicate clinical outcome. To test this idea, we devised an Epigenetic Instability Index (EpI) to evaluate instability for each tumour sample as the fraction of significant DNA methylation changes relative to a corresponding normal reference profile (Materials and Methods). The instability index was divided into 4 types according to the baseline normal reference methylation (0 = unmethylated, 1 = hemimethylated, 2 = methylated) and the nature of DNA methylation changes (0→1/2, 1→2, 1→0, 2→0/1) observed in cancer (Materials and Methods, Figure 3A and 3B). In addition, we considered the EpI restricted to PCGT and MESC stem cell loci, and since very few PCGT CpGs were observed to be methylated (1 or 2) in normal tissue, this resulted in 3 stem cell EpI indices: PCGT (0→1/2), MESC (1→0), MESC (2→0/1). Remarkably, we observed that the demethylation instability index (DeMI) at MESCs (2→0/1) was associated with poor prognosis in endometrial, breast, ovarian, and cervical cancers (Figure 4). In multivariate analysis, the DeMI was a predictor of poor prognosis in all cancers independently of other prognostic factors (Table 2 and Table S3), demonstrating the clinical potential of this DNA methylation stem cell signature. In contrast, the methylation instability index defined at PCGTs only correlated with clinical outcome in ovarian cancer (Table S3). Survival analysis at individual CpG level further demonstrated the consistent enrichment of MESC CpGs among prognostic CpGs hypomethylated in poor outcome samples in all 4 invasive cancers, whereas PCGT CpGs were not consistently enriched in either the hyper or hypomethylated prognostic component (Table S4). There was also substantial overlap between the MESC CpGs which have stable methylation levels in normal tissue and which become hypomethylated in cancer, and prognostic MESC CpGs that are hypomethylated in poor outcome tumour samples (Table S5).

**Table 2:** Survival analysis results of the PCGTs and MESCs Epigenetic Instability Index (EpI) in women's cancers.

	Univariate			Multivariate		
PCGT EpI	HR (95%CI)	P	n	HR (95%CI)	P	n
Endo CA	0.80 (0.48–1.33)	0.385	64	0.90 (0.53–1.54)	0.71	63
Ovarian CA	1.34 (1.13–1.59)	7.00E-04	177	1.54 (1.24–1.92)	9.00E-05	161
Cervical CA	0.75 (0.48–1.19)	0.222	48	0.96 (0.59–1.56)	0.878	47
Breast CA	0.99 (0.76–1.29)	0.949	113	0.93 (0.70–1.25)	0.645	107

MESC EpI (DeMI)	HR (95%CI)	P	n	HR (95%CI)	P	n
Endo CA	1.96 (1.30–2.96)	0.001	64	2.18 (1.24–3.83)	0.007	63
Ovarian CA	1.27 (1.05–1.54)	0.015	177	1.40 (1.09–1.79)	0.008	161
Cervical CA	1.42 (0.95–2.14)	0.086	48	1.47 (0.95–2.28)	0.085	47
Breast CA	1.53 (1.24–1.89)	5.00E-05	113	1.51 (1.20–1.89)	5.00E-04	107

Univariate and multivariate Cox regression results for the PCGT EpI and MESC EpI (DeMI) in endometrial, ovarian, cervical and breast cancer with number of samples (n), Hazard ratio (HR), 95% confidence interval (CI), and P-value (P). Overall survival was used for endometrial, ovarian and breast cancer, relapse free survival for cervical cancer.

**Figure 5:**
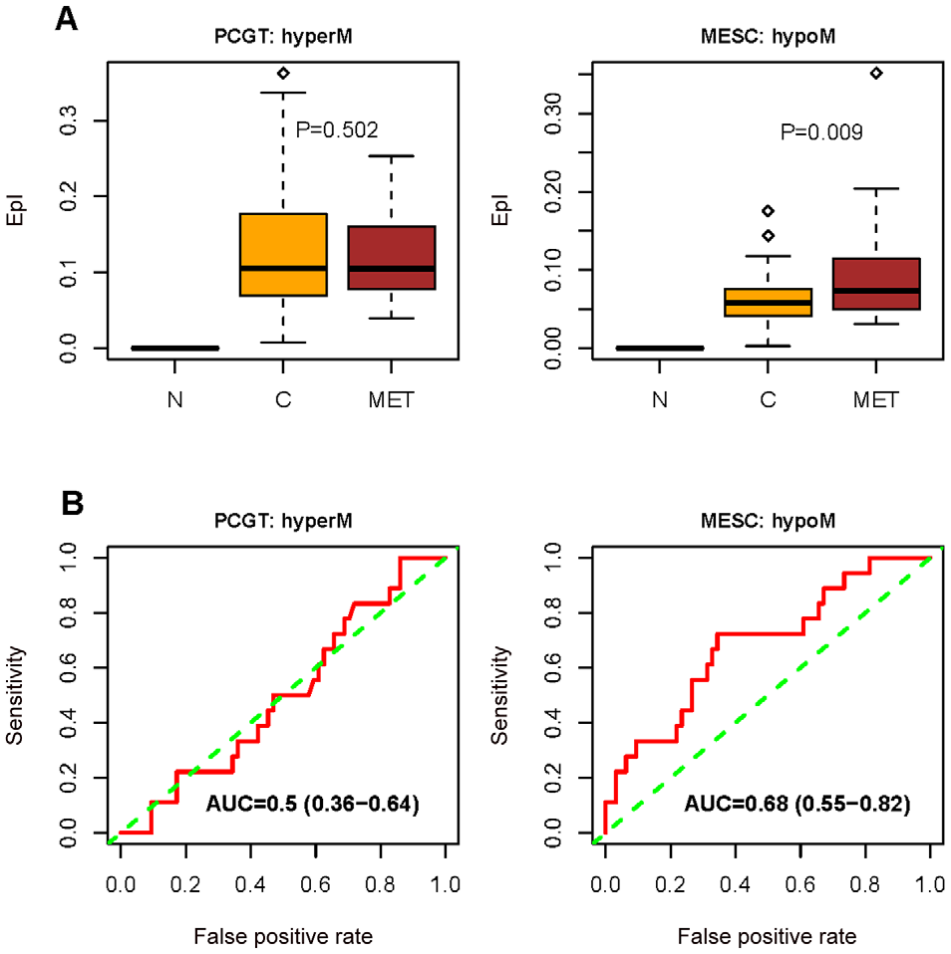
Methylation changes between normal, primary, and metastatic endometrial cancer. (A) Boxplots comparing the frequency of PCGT (0→1/2) DNA methylation changes, and the frequency of combined MESC (1→0) and MESC (2→0/1) DNA methylation changes (“combined DeMI index”), between normal endometrium (N), primary endometrial cancer (C), and between normal endometrium and metastatic endometrial cancers (MET). One-tailed Wilcoxon rank sum test P-values for the instability indices between cancer and metastases are indicated. (B) Receiver operating characteristic (ROC) curves measuring the dissimilarity in the combined DeMI index between primary and metastatic endometrial cancers with corresponding Area Under Curve (AUC) and 95% CI.

To further demonstrate that MESC hypomethylation is an important determinant of poor outcome in human cancer, we tested if these epigenetic changes progress further in metastatic cancer. Thus, we compared DNA methylation profiles of primary endometrial cancers to extra-uterine metastases of endometrial cancer. Importantly, the DeMI index was higher in metastatic cancer compared to primary tumours, but not so for the hypermethylation instability index at PCGTs (Figure 5A). In fact, the DeMI index demonstrates clinical potential for discriminating primaries that may be destined to metastasize (Figure 5B). From these data we can therefore conclude that while PCGT hypermethylation is an important event in early oncogenesis, which persists at later stages, MESC hypomethylation is a progressive process and a key characteristic of more malignant cancers (Figure 3B).

**Figure 6:**
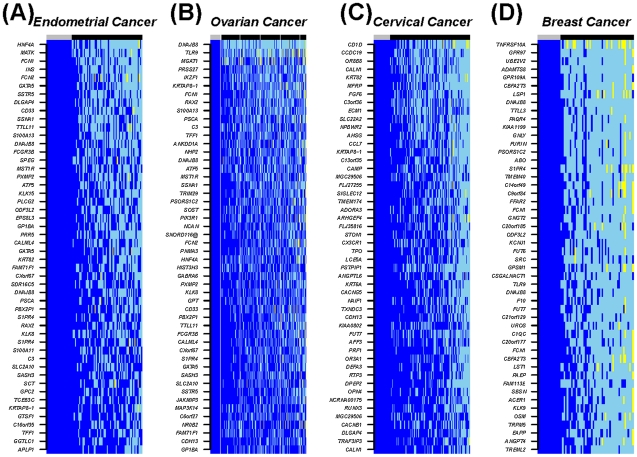
Heatmap of the top 50 most frequently hypomethylated MESCs in cancers. The CpGs show stable fully methylated states across all normal samples. Methylation values: blue = high methylation (β-value>0.7), skyblue = hemi methylation (0.25<β-value<0.7), yellow = low methylation (β-value<0.25). Sample labels at the top of the heatmaps: normal (grey) and cancer (black).

The ability of the DeMI index to predict clinical outcome in multiple cancers indicates that a core set of MESC CpGs may be involved. To investigate this we ranked the MESC CpGs according to the frequency of hypomethylation in each of the cancers considered. Many CpGs were observed to be hypomethylated in large fractions of tumours (Figure 6 and Table S6). While there were 6 MESC CpGs (*FCGR3B*, *FLJ27255*, *FCN2, KRT82*, *CDH13*, *KRTAP8-1* on chromosome 1, 6, 9, 12, 16 and 21 respectively) commonly hypomethylated at a frequency of at least 10% in all four cancers (P<10^−4^), there were substantially larger overlaps between related cancers such as ovarian and endometrial cancer (overlap of 98 CpGs, OR = 134, 95%CI = (89–205), P = 3.2×10^−124^). Gene Set Enrichment Analysis (GSEA) [26] of the hypomethylated MESCs in each cancer also revealed a striking overlap of enriched terms, especially between endometrial and ovarian cancer where we observed widespread hypomethylation at 20q11 and 9q34 (Table S7).

**Figure 7:**
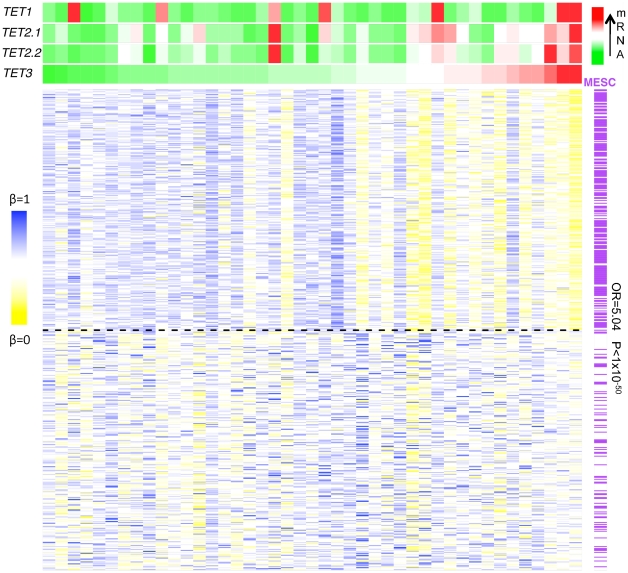
Anti-correlation between the TET mRNA expression level and methylation β-value. Heatmap of the 250 most (upper half) and least (lower half) anti-correlated hypomethylated CpGs in cervical cancer samples ranked according to their TET3 mRNA expression levels from the lowest (green) to the highest (red) with the indication of MESCs (purple) and nonMESCs (white). The odds ratio (OR) and P-value (P) are obtained from Fisher's exact test estimating the enrichment of MESCs among hypomethylated CpGs that are significantly anti-correlated with TET3 mRNA expression level.

Up until recently it has been assumed that DNA demethylation in cancer is a passive event, occurring as a result of absent re-methylation during DNA replication, with a consequent dilution of this covalent DNA modification. This view has now been substantially challenged by the identification of TET (ten eleven translocation) dioxygenases, which can convert 5-methylcytosine into 5-hydroxymethylcytosine and 5-carboxylcytosine, which thus constitutes a pathway for active DNA demethylation [13]–[17], [27]. In particular, it has been demonstrated that TET3-mediated DNA hydroxylation is involved in epigenetic reprogramming of the zygotic paternal DNA following natural fertilization and that this may also contribute to somatic cell nuclear reprogramming during animal cloning [16]. We therefore analysed mRNA expression of TET1 and two isoforms of TET2, and TET3 (see Text S1 for primer information), to test whether hypomethylation is associated with TET expression. We observed a strong correlation between high TET, in particular TET3 expression, and hypomethylation, specifically at MESC CpGs (Figure 7 and Figure S8). We checked that the anti-correlation of TET expression with MESC CpG methylation was independent of the level of methylation in normal tissue (Figure S9). Although this observation is purely correlative, it is consistent with the view that TET3 overexpression (Figure S10) in cancer contributes to reprogramming of cancer cells via active DNA demethylation.

## Discussion

Epithelial cells of the uterine cervix offer a unique opportunity to study epigenetic alterations throughout carcinogenesis. Our first key result is the demonstration that normal cells of origin acquire methylation changes at least three years in advance of the first morphological changes. Specifically, our data demonstrate that PCGT hypermethylation and MESC hypomethylation are major contributors to early cervical carcinogenesis. This is independent of human papillomavirus (HPV) infection as our study was matched for HPV status, and since PCGT enrichment was observed in both HPV+ and HPV− samples. Importantly, the observed enrichments were also independent of the levels of methylation in normal tissue. That is, MESCs which showed full methylation (i.e. β-value>0.8) or hemi-methylation (i.e. 0.3<β-value<0.7) were preferentially hypomethylated in dysplasia and cancer in comparison to control sets of CpGs with same methylation levels in normal tissue.

The role of PCGT methylation as a very early event is further supported by our finding that PCGTs were highly enriched among CpGs which were hypermethylated in blood cells from *BRCA1* mutation carriers, suggesting that *BRCA1* is an important regulator of the DNA methylome and that aberrant *BRCA1* function could lead to increased predisposition to cancer through increased methylation at PCGT loci. The fact that *BRCA1* mutation carriers showed increased PCGT methylation in their blood cells but are at no substantial increased risk to develop blood-borne cancers suggests that PCGT hypermethylation refers a substantial risk but that there are additional factors required (e.g. endocrine, paracrine or viral triggers).

Our second key result is that MESC hypomethylation occurs in both the epithelial and stromal components of cancer and that this is a progressive process, increasing significantly towards invasion and metastatic cancer. This in turn suggests that the level of MESC hypomethylation in primary tumours may be an important determinant of clinical outcome.

Indeed, our third key result is the report of a stem cell (MESC) DNA hypomethylation signature that can predict clinical outcome in multiple human cancers, independently of known prognostic factors. To the best of our knowledge this constitutes the first report of a common prognostic signature in cancer that is based on DNA methylation, and is therefore an epigenetic analogue to the prognostic genomic instability signature presented in [3].

Besides the key distinction of PCGT and MESC CpGs, we also observed that the localisation of CpGs in relation to PMDs was another important facet of the pattern of DNA methylation changes. Specifically, PCGT hypermethylation was observed preferentially within PMDs, while the progressive MESC hypomethylation in cancer was equally strong in PMDs and non-PMDs. We point out that while the PMDs considered here were defined for colon cancer cells, that these broad regions of partial methylation overlap significantly between colon tissue and fibroblasts, suggesting that these regions may be largely similar also between different tissues.

The similarities between normal developmental and cancer epigenetic programming are intriguing. While embryonic stem cells suppress differentiation-inducing genes reversibly via promoter occupancy of PRC2, cancer cells suppress these same genes much more robustly via covalent DNA modification. Even more interestingly, trophoblast cells whose core function is to invade the maternal tissue and form the placenta, are relatively more hypomethylated compared with the inner cell mass, which will differentiate into the embryo [28], supporting the view that hypomethylation may be associated with the capacity to invade neighbouring tissue such as the maternal endometrium. Similarly, the observed correlation between MESC hypomethylation and the malignant potential of cancers suggests that fully methylated MESCs may provide a protective mechanism against invasion. Thus, the fact that the great majority of MESCs exhibit similar high methylation levels in stem cells and normal tissues, means that high MESC methylation may be viewed as an intrinsic property of any normal cell, regardless of whether it is a stem cell or a mature differentiated one. In this model then, hypomethylation at MESCs would lead to a transformed cellular phenotype that is more prone to invasion. In this context however, it is worth pointing out that the observed MESC hypomethylation could also be reflecting changes in the stromal cell content of the tumours. Indeed, the observation that cancer fibroblasts show similar hypomethylation changes at MESC loci suggests that the more frequent MESC hypomethylation in invasive cancers could be partly due to increased numbers of cancer fibroblasts.

It could also be argued that the other DNA methylation changes we have reported here are the result of changes in the stromal and immune cell compartments of the tumours. However, we verified using Principal Components Analysis (PCA) and GSEA analysis [26] on normal liquid based cytology (LBC) samples and separately on age-matched cervical dysplasias (Table 1, “Dy”-study) that the components of variation associated with stromal and immune cell markers were very similar between normal and dysplasia, in stark contrast to PCGTs which showed a dramatic difference with comparatively no variation in normal tissue but representing the dominant component of variation in dysplasia (manuscript in preparation). Thus, the DNA methylation changes at PCGT loci reported here are unlikely to be due to changes in the stromal cell composition of tumours.

Finally, the crucial role of TET3 in DNA demethylation and early development, its overexpression in cancer, and the observed correlation with MESC hypomethylation, supports the view that aberrant developmental programs leading to reprogramming of the epigenome in adult cells may be critical for carcinogenesis. Interfering with these aberrant programs may therefore lead to novel ways to treat cancer.

In summary, our findings suggest that epigenetic deregulation of two distinct sets of genes, both important for stem cell integrity, impact carcinogenesis in different ways: one process involves gain of methylation and is a hallmark of de-differentiation and early oncogenesis, while the other involves loss of methylation and is a key determinant of invasion and clinical outcome.

## Materials and Methods

### Definition of MESC

A recent study used bisulfite sequencing to map, at single-base-resolution, DNA methylation throughout the majority of the human genome in both embryonic stem cells and fibroblasts [12]. For each CpG site, the number of C and T reads covering each methyl cytosine on both forward and reverse strands were provided [12]. The multiple reads covering each methyl cytosine can be used as readout of the fraction of sequences within the sample that are methylated at that particular site (i.e. C reads/C+T reads) [29], and hence, referred as the methylation level of the site. In this study, Methylated in human Embryonic Stem Cells (MESC) CpGs are the CpG sites that were covered by at least 5 reads on both forward and reverse strands (i.e. the total number of C and T reads on both strands > = 5) and the overall mean methylation levels (i.e. the average methylation level of both the forward and reverse strands) is greater than 80%. MESC CpGs were then mapped to those present on the Illumina 27 k array (Table S1). Functional annotation (gene assignment) of the MESC CpGs present on the array was obtained from Illumina and Bioconductor annotation packages.

### Definition of PCGTs

PolyComb Group Target genes (PCGTs) were defined as CpGs which are occupied by SUZ12 and/or EeD and/or are trimethylated at Lysine 27 on histone H3 in human embryonic stem cells (Table S2, annotation file kindly provided by Benjamin P. Berman and Peter W. Laird) [19].

### DNA Methylation Assay

#### DNA extraction

DNA from LBC samples and tissues was isolated using the Qiagen DNeasy Blood and Tissue Kit (Qiagen Ltd, UK, 69506) and quantified via spectrophotometry (Nanodrop, Thermo Scientific Ltd UK) with 600 ng DNA from each sample. DNA from whole blood was extracted using a chloroform based extraction method from 400 µL of blood. All DNA samples were bisulphite modified using the EZ DNA Methylation Kit D5004/8 (Zymo Research, Orange, CA, USA) according to the manufacturer's instructions.

#### DNA methylation profiling

The genome wide methylation analyses were performed using the validated Illumina Infinium Human Methylation27 BeadChip (Illumina Inc USA, WG-311-1201) [18]. During the assay, bisulphite (BS) converted DNA is amplified, fragmented and hybridised to the BeadChip arrays (each chip accommodates 12 samples as designated by Sentrix positions A–L). A single base extension is then performed using labelled DNP- and biotin labelled dNTPs. The arrays were imaged using a BeadArray Reader. Image processing and intensity data extraction were performed according to Illumina's instructions. Each interrogated locus is represented by specific oligomers linked to two bead types: one representing the sequence for methylated DNA (M) and the other for unmethylated DNA (U). For each specific CpG site, the methylation status is calculated from the intensity of the M and U alleles, as the ratio of the fluorescent signals β = Max(M,0)/[Max(M,0)+Max(U,0)+100]. Hence, DNA methylation β-values are continuous variables between 0 (absent methylation) and 1 (completely methylated) representing the ratio of the methylated allele to the combined locus intensity.

#### TET expression

Total RNA was isolated as previously described [30]. Reverse transcription of RNA was performed using M-MLV Reverse Transcriptase (Promega) according to the manufacturer's instructions. Primers and probes for the TET genes were designed using Primer Express (Applied Biosystems, Foster City, CA, USA). Samples in which TET was not amplified by real-time PCR after 45 cycles were classified as TET negative.

### Statistical Methods

#### Quality control and inter-array normalisation

Quality control procedures and intra-array normalisation were run on all data except the ‘Colon CA’, ‘Lung CA’, and ‘Ovarian CA’ sets, for which the intra-array normalised data was downloaded directly from Gene Expression Omnibus (GEO) and The Cancer Genome Atlas (TCGA) databases. Background corrected *U* and *M* values, β-values (as generated from the Beadstudio software) and built-in controls were used to evaluate the quality of individual arrays. Samples with low BS conversion efficiency (BS control intensity values <4000) were excluded, as well as other outliers that we detected using boxplots of total intensity *I* = *U*+*M* values and histograms of β-values. Samples were filtered further according to CpG coverage, using the Beadstudio P-values of detection of signal above background.

#### Enrichment analysis

Enrichment analysis was performed using a two-tailed Fisher's exact test. Odds ratios (OR) and 95% confidence intervals (CI) of enrichment were also computed and their corresponding significance levels estimated. Enrichment analysis was performed with a range of thresholds to check for robustness and using the Infinium 27 k array as reference to avoid array-specific bias.

#### Supervised analysis

A linear regression approach was used to model the association between disease status (cases or controls) and the CpG β-value methylation profile. Adjustment for age and experimental factors (e.g. bisulphite conversion) was performed by inclusion of these factors in the model as covariates. Chip effects were observed, and in this study all data were adjusted by either applying the “ComBat” method (a method that is robust to outliers and that allows for adjustment in cases where sample sizes per chip are small) [31] or using the chip as a covariate in the linear model. The linear model was adopted over a non-linear logistic or probit model as the linear model performed better in capturing profiles with larger effect sizes.

#### Skewness analysis

Given the two disease-status-associated CpG lists (hyper- or hypomethylated) obtained from the supervised analysis, the two-tailed binomial test was used to detect the skewness of the methylation in various categories (i.e. colon-PMD PCGTs, colon-PMD MESCs, nonPMD PCGTs, and nonPMD MESCs) of the CpGs (Figure 2, Figures S1 and S2).

#### Epigenetic instability analysis

We devised an Epigenetic Instability Index (EpI) for each tumour sample as follows. First, CpG readings were defined as unmethylated (0) (β-value<0.25), hemimethylated (1) (0.25≤β-value≤0.7), and methylated (2) (β-value>0.7). Next, we selected CpGs with stable methylation profiles in normal tissue, defined as those CpGs with the same methylation state in all normal samples corresponding to the given tissue. These stable CpGs can undergo four types of DNA methylation changes in cancer: 0->1/2, 1->2, 1->0 and 2->0/1. Therefore, for each tumour sample, we computed four different “instability” indices, reflecting the fraction of stable CpGs undergoing the specific types of DNA methylation changes shown. When computing these indices, and to ensure their robustness to the choice of methylation thresholds above, we also required at least a 10% change in β-values for calling DNA methylation differences between normal and cancer tissue. This buffering therefore avoids calling potentially small differences in β-values (<10%), which nevertheless may trespass the methylation thresholds (0.25, 0.7) used. The EpI indices were also computed by restricting the set of stable CpGs to those mapping to PCGT and MESC stem cell loci. Since the great majority of PCGT CpGs were observed to be stably unmethylated (0) in normal tissue, this resulted in 3 “stem cell EpI” indices: PCGT (0->1/2), MESC (1->0), MESC (2->0/1). We call the latter index the Demethylation instability index (DeMI).

#### Survival analysis

Univariate and multivariate Cox regression models were used for the survival analysis. In the multivariate analysis, besides DNA methylation β-values (or the EpI index), those clinical and histological factors, which were associated with survival in univariate analysis were also included as covariates.

## Supporting Information

Figure S1 Differential dynamics of hypermethylated and hypomethylated PMD PCGTs and PMD MESCs. Bar charts representing percentages of significantly hypermethylated (blue) and hypomethylated (orange) PMD PCGT and PMD MESC CpGs in (A) each stage of cervical carcinogenesis: Cervix ‘Before Dysplasia’, ‘Dysplasia’, and ‘Invasive Cancer’, all relative to normal cervix tissue; and in (B) ‘Breast CA’, ‘Endo CA’, ‘Colon CA’, and ‘Lung CA’, all relative to their respective normal controls. The significance of the binomial test assessing skew of hypermethylated versus hypomethylated ( and S4) is indicated by ‘*’, ‘**’, and ‘***’ for P-value<0.05, 0.01, and 0.001 respectively.
(TIF)

Figure S2 Differential dynamics of hypermethylated and hypomethylated nonPMD PCGTs and nonPMD MESCs. Bar charts of the percentages between the disease (or mutation) status associated hypermethylated (blue) and hypomethylated (orange) for nonPMD polycomb group target gene (PCGT) CpGs and nonPMD methylated in human embryonic stem cells (MESC) CpGs that pass their corresponding significance level thresholds (the same notation as in Figure S1).
(TIF)

Figure S3 Statistical output from linear regression model estimating the association of the PMD PCGT and PMD MESC CpGs to the outcomes of the three stages of cervical carcinogenesis. Scatterplots of the linear regression fitted (adjusted for age, chip and bisulphite conversion) t-statistics against their corresponding −log_10_(P-values) that test the association with the cases and controls of the cervix ‘Before Dysplasia’ (CIN2/3 status), ‘Dysplasia’ (CIN2/3 status), and ‘Invasive Cancer’ (cancer status) on the PMD PCGT (left column) and PMD MESC (right column) CpGs. Green vertical lines denote the significant level thresholds of P-value = 0.1 for ‘Before Dysplasia’ and ‘Dysplasia’, and 0.001 for ‘Invasive Cancer’. The overall numbers of CpGs that are hypermethylated (blue) and hypomethylated (orange) with their associated two-sided Binomial test P-value are given on the left hand side of the P-value threshold lines and the number of CpGs that are hypermethylated (blue) and hypomethylated (orange) pass the corresponding P-value threshold with their Binomial test P-value on the right.
(TIF)

Figure S4 Statistical output from linear regression model estimating the association of the nonPMD PCGT and nonPMD MESC CpGs to the outcomes of the three stages of cervical carcinogenesis. Scatterplots of three cervical sets, similar to Figure S3, but based on the nonPMD PCGT (left column) and nonPMD MESC (right column) CpGs.
(TIF)

Figure S5 Enrichment analysis of PMD PCGTs and PMD MESCs in the hyper- and hypomethylated cervical cancer CpGs. Cumulative enrichment analysis (Fisher's exact tests ORs and P-values) of PCGTs among CpGs unmethylated (mean β-value<0.2 in normal cervix) in normal cervix and which become hypermethylated in (i) normal samples three years prior to dysplasia (BDy), (ii) non-invasive dysplastic samples (Dy), and (iii) invasive cervical cancer (CA) in PMDs (A) and nonPMDs (B) respectively. Similarly, enrichment of MESCs among CpGs methylated (mean β-value>0.4 in normal cervix) in normal cervix and that become hypomethylated in cases in PMDs (C) and nonPMDs (D) respectively.
(TIF)

Figure S6 Statistical output from linear regression models estimating the association of the PMD PCGTs and PMD MESC CpGs to outcomes in five cohorts. Scatterplots of the linear regression fitted (adjusted for age, chip and bisulfite conversion) t-statistics against their corresponding −log_10_(P-values) testing the association with the cases and controls of ‘Breast CA’ (cancer status), ‘Endo CA’ (cancer status), ‘Colon CA’ (cancer status), ‘Lung CA’ (cancer status), and ‘BRCA1 MUT’ (BRCA1 status) on the PMD PCGT and PMD MESC CpGs. Green vertical lines denote the significant level thresholds of P-value = 0.1 for ‘BRCA1 MUT’ and 0.001 for all the others. The overall number of CpGs that are hypermethylated (blue) and hypomethylated (orange) with their associated two-sided Binomial test P-value are given on the left hand side of the P-value threshold lines. The number of CpGs that are hypermethylated (blue) and hypomethylated (orange) pass the corresponding P-value threshold with their Binomial test P-values on the right.
(TIF)

Figure S7 Statistical output from linear regression models estimating the association of the nonPMD PCGT and nonPMD MESC CpGs to the outcomes in five cohorts. Scatterplots of five cohorts, similar to Figure S6, based on the nonPMD PCGT (left column) and nonPMD MESC (right column) CpGs.
(TIF)

Figure S8 Magnitude of the anti-correlation between hypomethylated cervical cancer CpGs and TET mRNA. Hypomethylated MESCs are significantly higher anti-correlated with TET2 and TET3 mRNA expression levels than the hypomethylated nonMESCs in the cervical cancer samples. P-values are obtained from the Wilcoxon one-sided test.
(TIF)

Figure S9 Magnitude of the anti-correlation between hypomethylated cervical cancer CpGs (grouped) and TET mRNA. Higher anti-correlated signature with TET2 and TET3 mRNA expression levels among hypomethylated MESCs than hypomethylated nonMESCs in the cervical cancer samples independent from the chosen baselines of the methylated and hemimethylated CpGs (mean β-value in normals >0.4). P-values are obtained from the Wilcoxon one-sided test.
(TIF)

Figure S10 TET mRNA expression level comparison between the normal cervix and cervical cancers. Boxplots of TET1, TET2.1, TET2.2 and TET3 mRNA expression levels of the normal cervix and cervical cancers. P-values are obtained from the Wilcoxon two-sided test.
(TIF)

Table S1 The MESC CpGs mapped to 27 k Infinium array. List of the 5,943 MESC CpGs that mapped to the Illumina Infinium Human Methylation27 beadchip array with the information of their IlluminaID, geneID, gene symbol, MapInfo, and chromosome.
(XLS)

Table S2 The PCGT CpGs mapped to 27 k Infinium array. Similar to Table S1, the list of the 3,465 PCGT CpGs that mapped to Illumina Infinium Human Methylation27 beadchip array.
(XLS)

Table S3 Summary tables of the uni- and multivariate Cox regression model analysis of the PCGTs and MESCs Epigenetic Instability Index (EpI) in endometrial, breast, ovarian, and cervical cancers. Univariate (UV) and multivariate (MV) Cox regression results for the PCGTs and MESCs EpI in endometrial, breast, ovarian and cervical cancer overall survival (OS) and relapse free survival (REL) with number of samples (n), Hazard ratio (HR), 95% confident interval, and P-value (P).
(XLS)

Table S4 PCGT and MESC enrichment analysis amongst hypermethylated and hypomethylated cancer prognostic CpGs. Enrichment analysis (Fisher's exact tests odds ratios (OR), 95% confidence intervals (CI) and P-values (P)) of PCGTs (colon-PMD and nonPMD) and MESCs (colon-PMD and nonPMD) among the top 500 hypermethylated and hypomethylated cancer (cervical, breast, endometrial, and ovarian cancers respectively) prognostic CpGs.
(XLS)

Table S5 Overlap between MESC CpGs with stable methylation levels in normal tissue and that become hypomethylated in cancer with the top ranked 1,000 prognostic MESC CpGs that are hypomethylated in poor outcome samples in endometrial, breast, ovarian, and cervical cancers.
(XLS)

Table S6 MESC CpGs according to the frequency of hypomethylation (cancer vs normal) as defined by the demethylation (2->0/1) instability index (DeMI) in the endometrial, breast, ovarian, and cervical cancers.
(XLS)

Table S7 GSEA results of MESC CpGs with a frequency of hypomethylation (defined by DeMI index) in cancer of at least 5% (endometrial, breast, and ovarian cancer) and of at least 10% in cervical cancer.
(XLS)

Text S1 A detailed description of the definitions, study population, materials, and primers used in this study is provided.
(DOC)
