# Correction: The Dynamics and Prognostic Potential of DNA Methylation Changes at Stem Cell Gene Loci in Women's Cancer

**DOI:** 10.1371/annotation/35f168f3-c509-4b4f-b245-f6682325838e

**Published:** 2012-03-23

**Authors:** Joanna Zhuang, Allison Jones, Shih-Han Lee, Esther Ng, Heidi Fiegl, Michal Zikan, David Cibula, Alexandra Sargent, Helga B. Salvesen, Ian J. Jacobs, Henry C. Kitchener, Andrew E. Teschendorff, Martin Widschwendter

The number of samples quoted in the BC and EC rows are incorrect. Please view the correct Table 1 here: 

**Figure:**
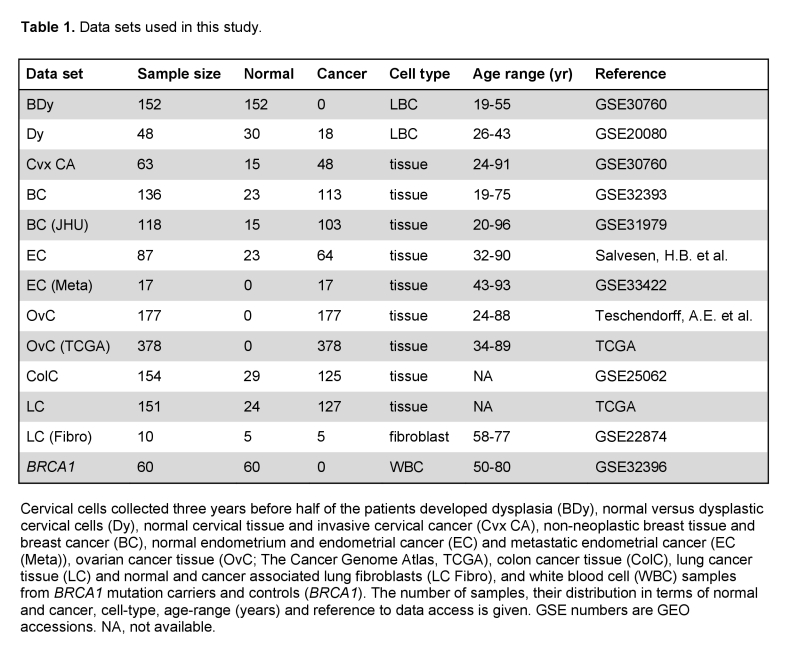


.

