# Supplementary figures and images for: The Dynamics and Prognostic Potential of DNA Methylation Changes at Stem Cell Gene Loci in Women's Cancer
Source: PLoS Genet. 2012 Feb 9;8(2):e1002517. doi: 10.1371/journal.pgen.1002517 (PMC3276553; doi:10.1371/journal.pgen.1002517)

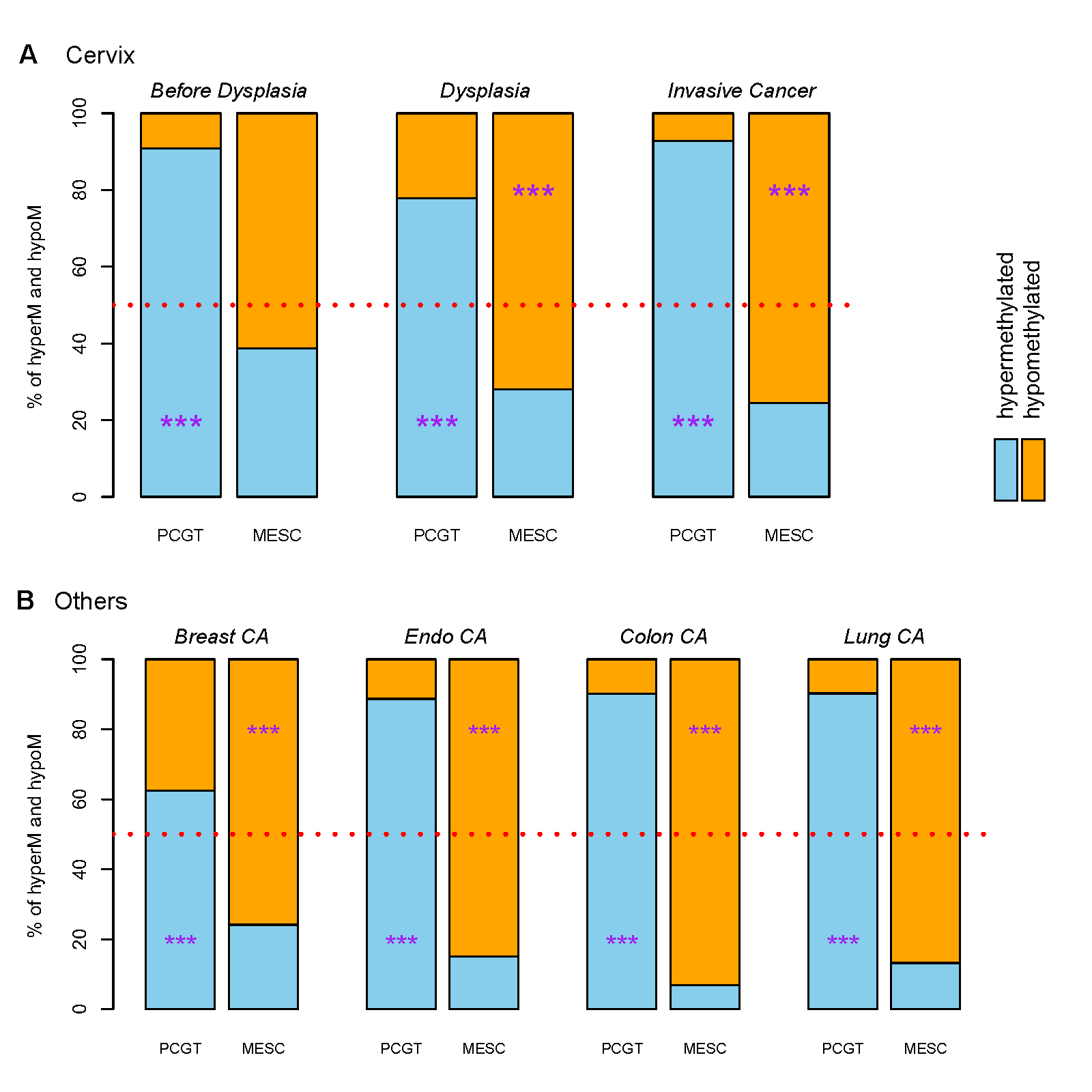

Supplement: Figure S1 — Differential dynamics of hypermethylated and hypomethylated PMD PCGTs and PMD MESCs. Bar charts representing percentages of significantly hypermethylated (blue) and hypomethylated (orange) PMD PCGT and PMD MESC CpGs in (A) each stage of cervical carcinogenesis: Cervix ‘Before Dysplasia’, ‘Dysplasia’, and ‘Invasive Cancer’, all relative to normal cervix tissue; and in (B) ‘Breast CA’, ‘Endo CA’, ‘Colon CA’, and ‘Lung CA’, all relative to their respective normal controls. The significance of the binomial test assessing skew of hypermethylated versus hypomethylated ( and S4) is indicated by ‘*’, ‘**’, and ‘***’ for P-value<0.05, 0.01, and 0.001 respectively. (TIF) [file pgen.1002517.s001.tif]

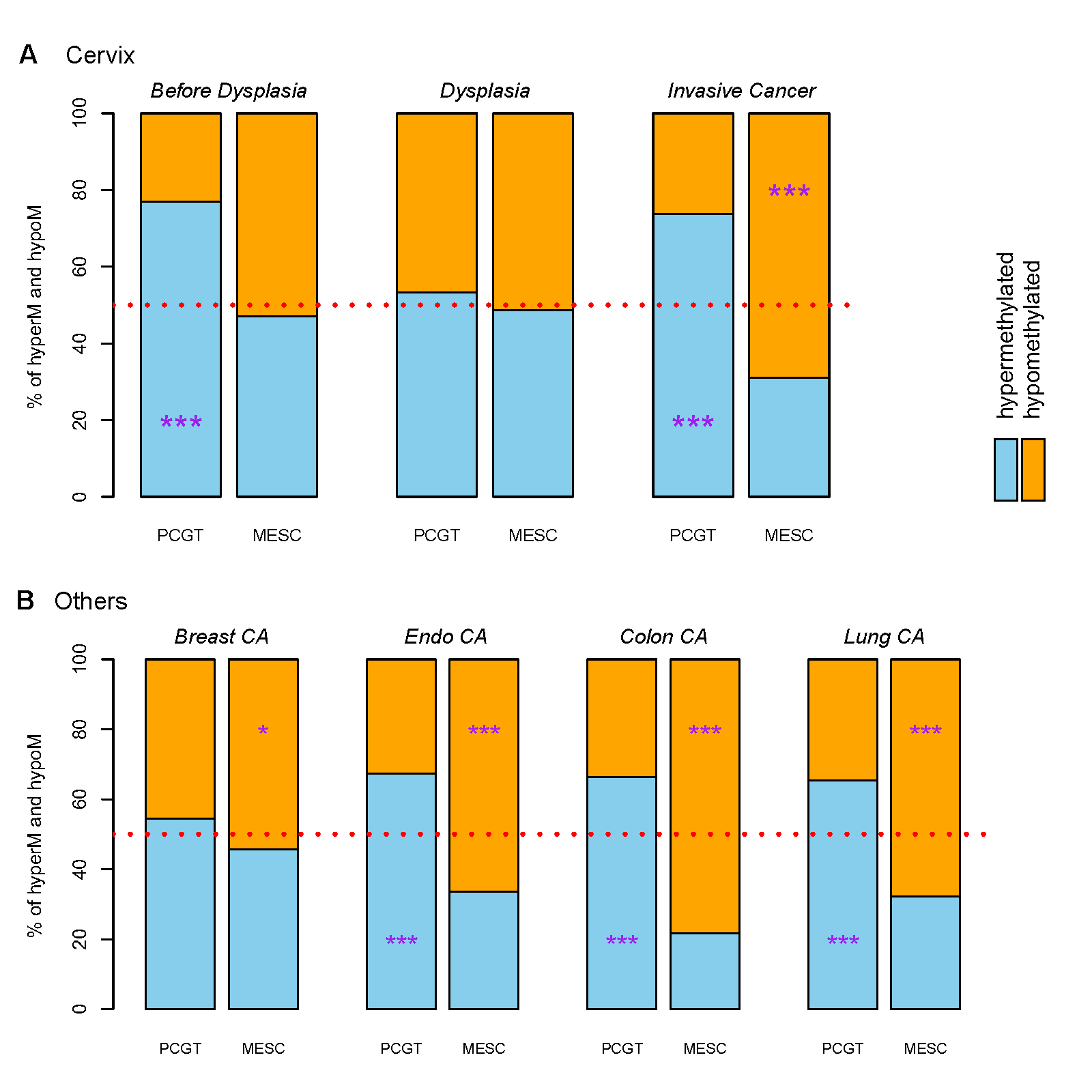

Supplement: Figure S2 — Differential dynamics of hypermethylated and hypomethylated nonPMD PCGTs and nonPMD MESCs. Bar charts of the percentages between the disease (or mutation) status associated hypermethylated (blue) and hypomethylated (orange) for nonPMD polycomb group target gene (PCGT) CpGs and nonPMD methylated in human embryonic stem cells (MESC) CpGs that pass their corresponding significance level thresholds (the same notation as in Figure S1). (TIF) [file pgen.1002517.s002.tif]

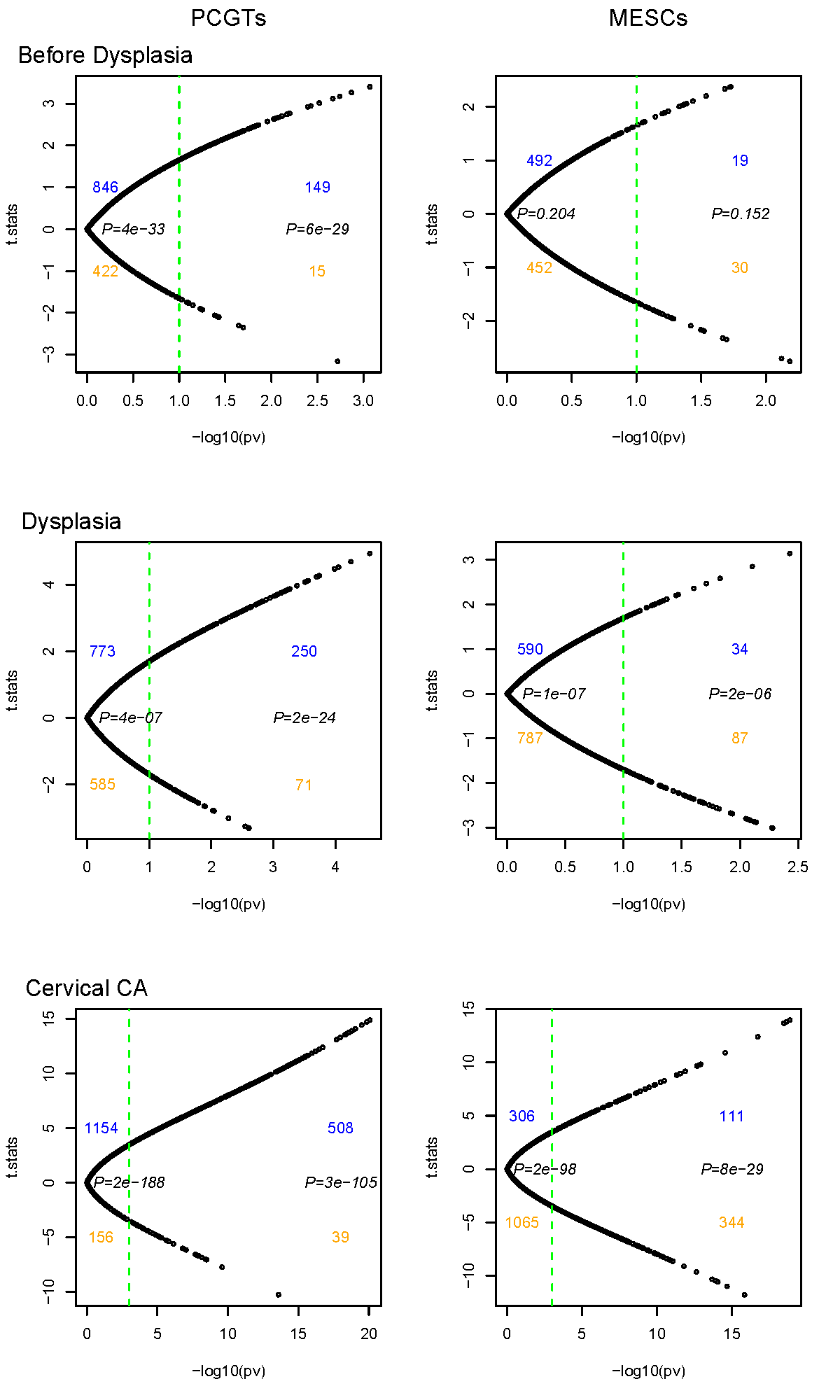

Supplement: Figure S3 — Statistical output from linear regression model estimating the association of the PMD PCGT and PMD MESC CpGs to the outcomes of the three stages of cervical carcinogenesis. Scatterplots of the linear regression fitted (adjusted for age, chip and bisulphite conversion) t-statistics against their corresponding −log10(P-values) that test the association with the cases and controls of the cervix ‘Before Dysplasia’ (CIN2/3 status), ‘Dysplasia’ (CIN2/3 status), and ‘Invasive Cancer’ (cancer status) on the PMD PCGT (left column) and PMD MESC (right column) CpGs. Green vertical lines denote the significant level thresholds of P-value = 0.1 for ‘Before Dysplasia’ and ‘Dysplasia’, and 0.001 for ‘Invasive Cancer’. The overall numbers of CpGs that are hypermethylated (blue) and hypomethylated (orange) with their associated two-sided Binomial test P-value are given on the left hand side of the P-value threshold lines and the number of CpGs that are hypermethylated (blue) and hypomethylated (orange) pass the corresponding P-value threshold with their Binomial test P-value on the right. (TIF) [file pgen.1002517.s003.tif]

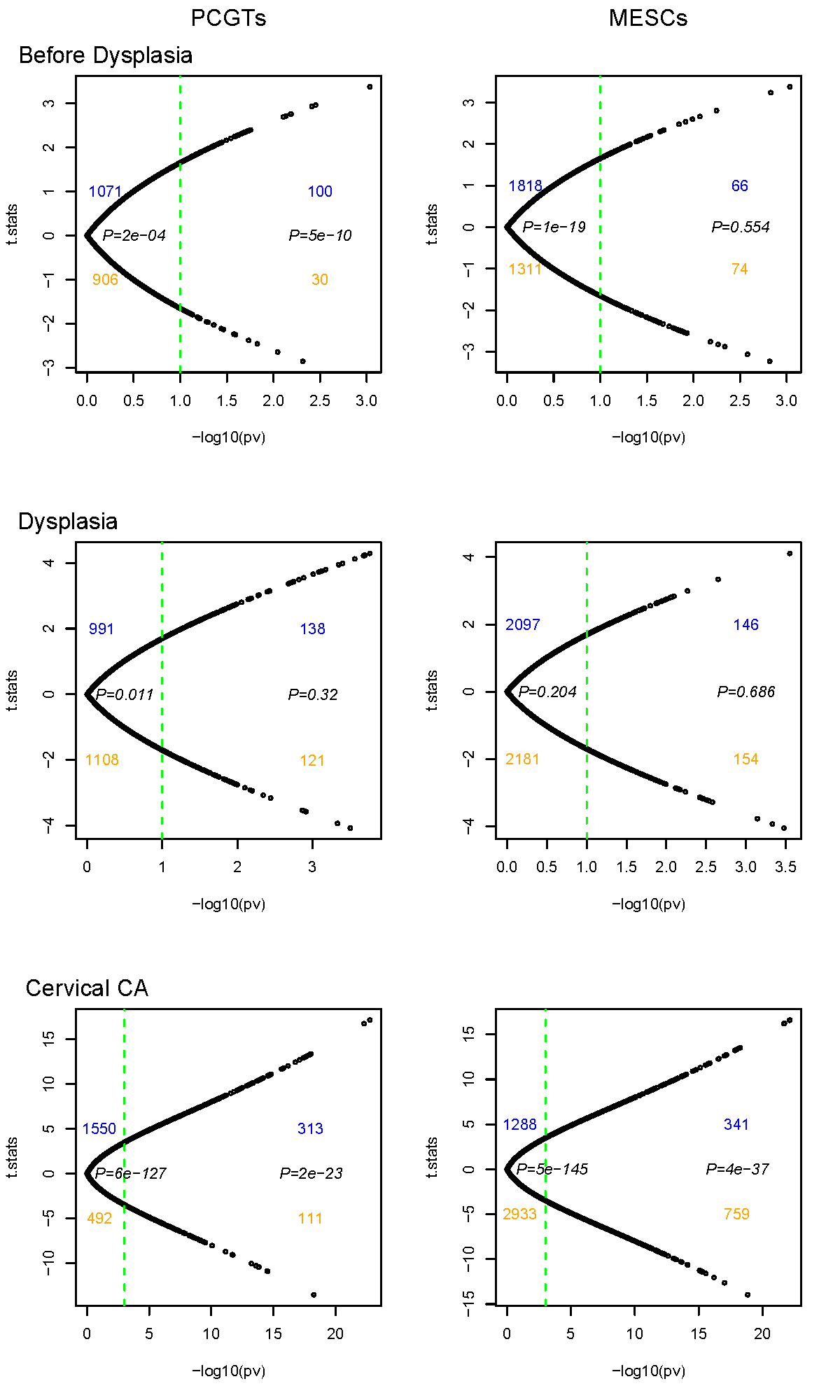

Supplement: Figure S4 — Statistical output from linear regression model estimating the association of the nonPMD PCGT and nonPMD MESC CpGs to the outcomes of the three stages of cervical carcinogenesis. Scatterplots of three cervical sets, similar to Figure S3, but based on the nonPMD PCGT (left column) and nonPMD MESC (right column) CpGs. (TIF) [file pgen.1002517.s004.tif]

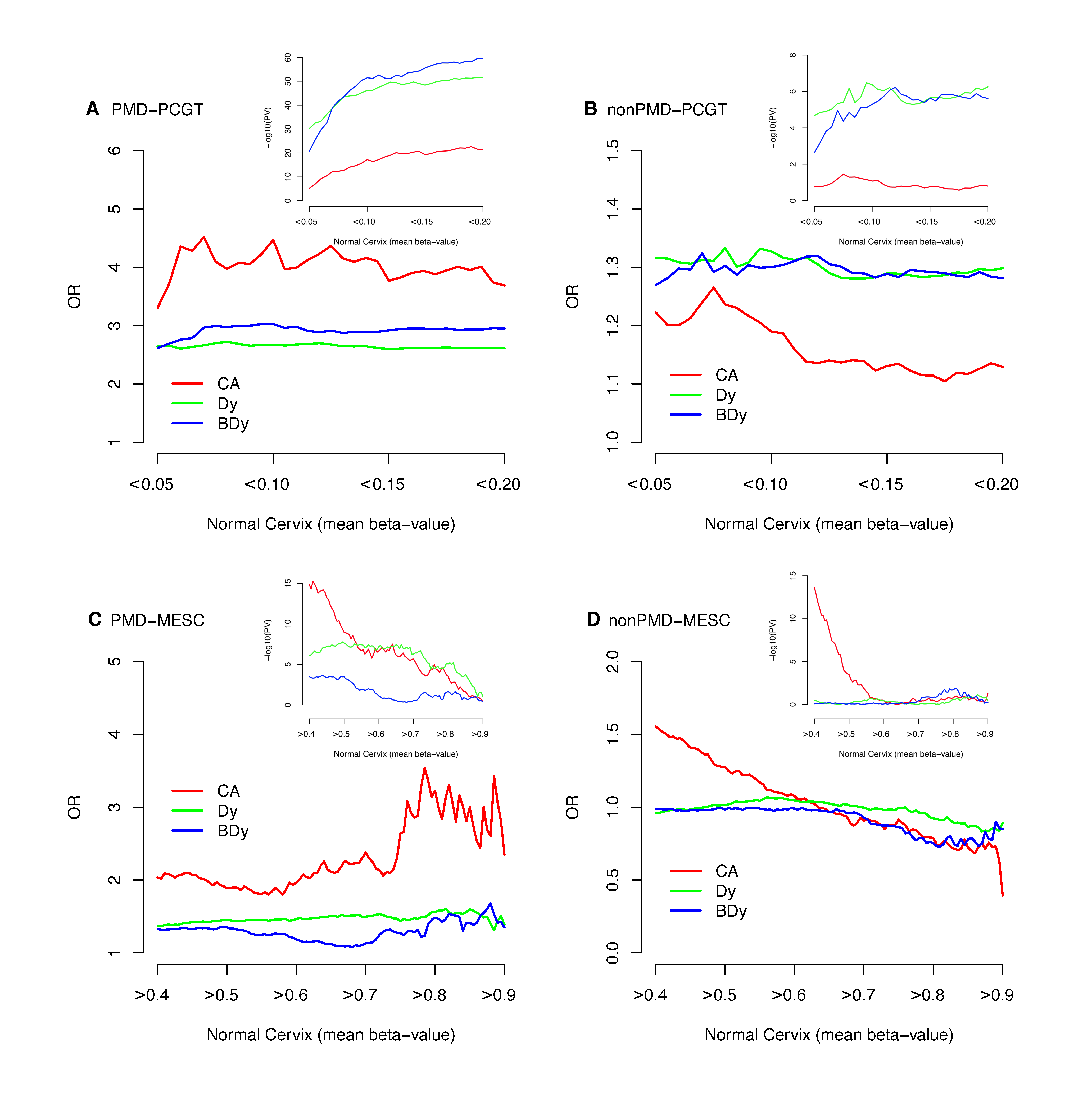

Supplement: Figure S5 — Enrichment analysis of PMD PCGTs and PMD MESCs in the hyper- and hypomethylated cervical cancer CpGs. Cumulative enrichment analysis (Fisher's exact tests ORs and P-values) of PCGTs among CpGs unmethylated (mean β-value<0.2 in normal cervix) in normal cervix and which become hypermethylated in (i) normal samples three years prior to dysplasia (BDy), (ii) non-invasive dysplastic samples (Dy), and (iii) invasive cervical cancer (CA) in PMDs (A) and nonPMDs (B) respectively. Similarly, enrichment of MESCs among CpGs methylated (mean β-value>0.4 in normal cervix) in normal cervix and that become hypomethylated in cases in PMDs (C) and nonPMDs (D) respectively. (TIF) [file pgen.1002517.s005.tif]

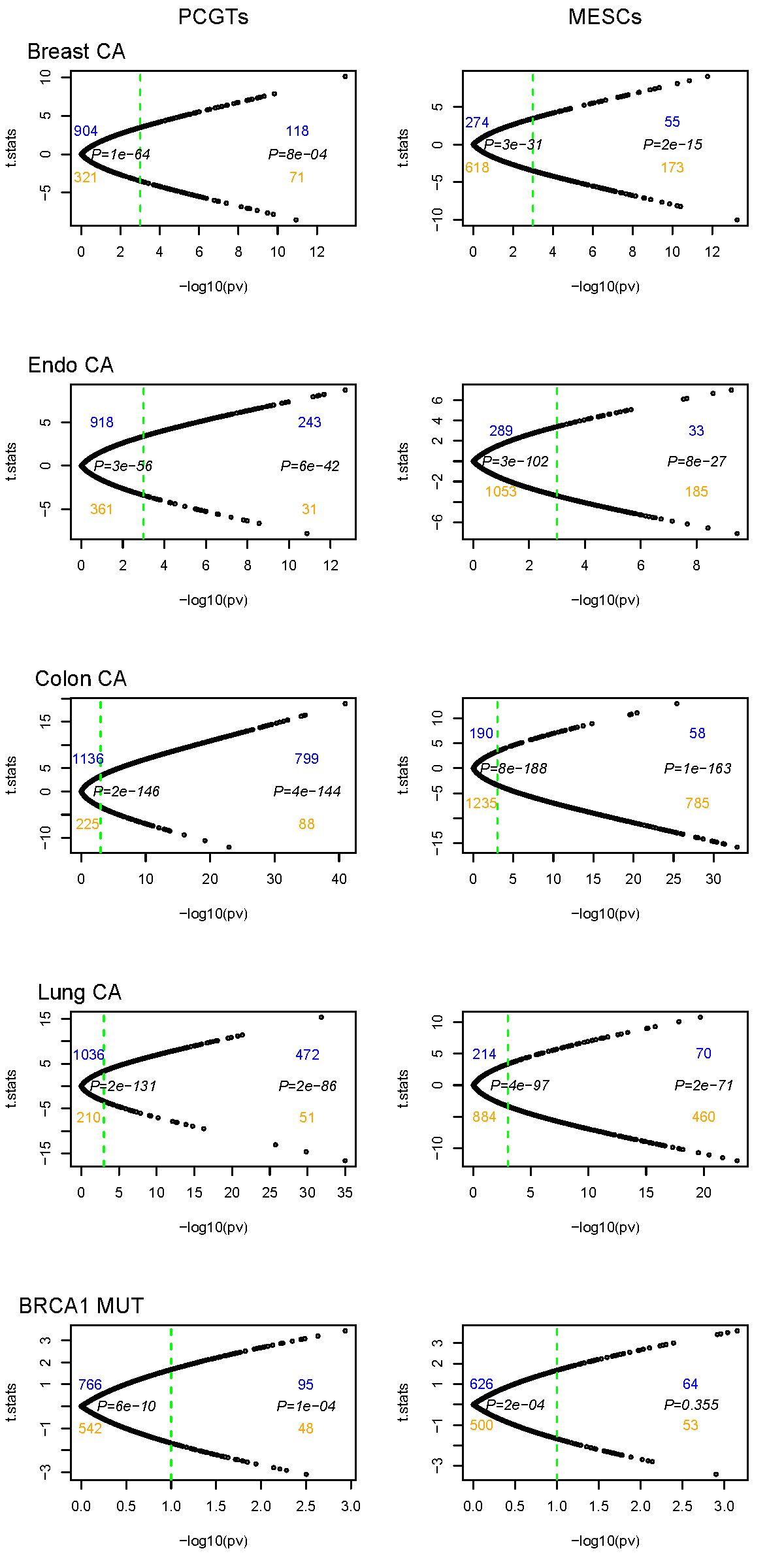

Supplement: Figure S6 — Statistical output from linear regression models estimating the association of the PMD PCGTs and PMD MESC CpGs to outcomes in five cohorts. Scatterplots of the linear regression fitted (adjusted for age, chip and bisulfite conversion) t-statistics against their corresponding −log10(P-values) testing the association with the cases and controls of ‘Breast CA’ (cancer status), ‘Endo CA’ (cancer status), ‘Colon CA’ (cancer status), ‘Lung CA’ (cancer status), and ‘BRCA1 MUT’ (BRCA1 status) on the PMD PCGT and PMD MESC CpGs. Green vertical lines denote the significant level thresholds of P-value = 0.1 for ‘BRCA1 MUT’ and 0.001 for all the others. The overall number of CpGs that are hypermethylated (blue) and hypomethylated (orange) with their associated two-sided Binomial test P-value are given on the left hand side of the P-value threshold lines. The number of CpGs that are hypermethylated (blue) and hypomethylated (orange) pass the corresponding P-value threshold with their Binomial test P-values on the right. (TIF) [file pgen.1002517.s006.tif]

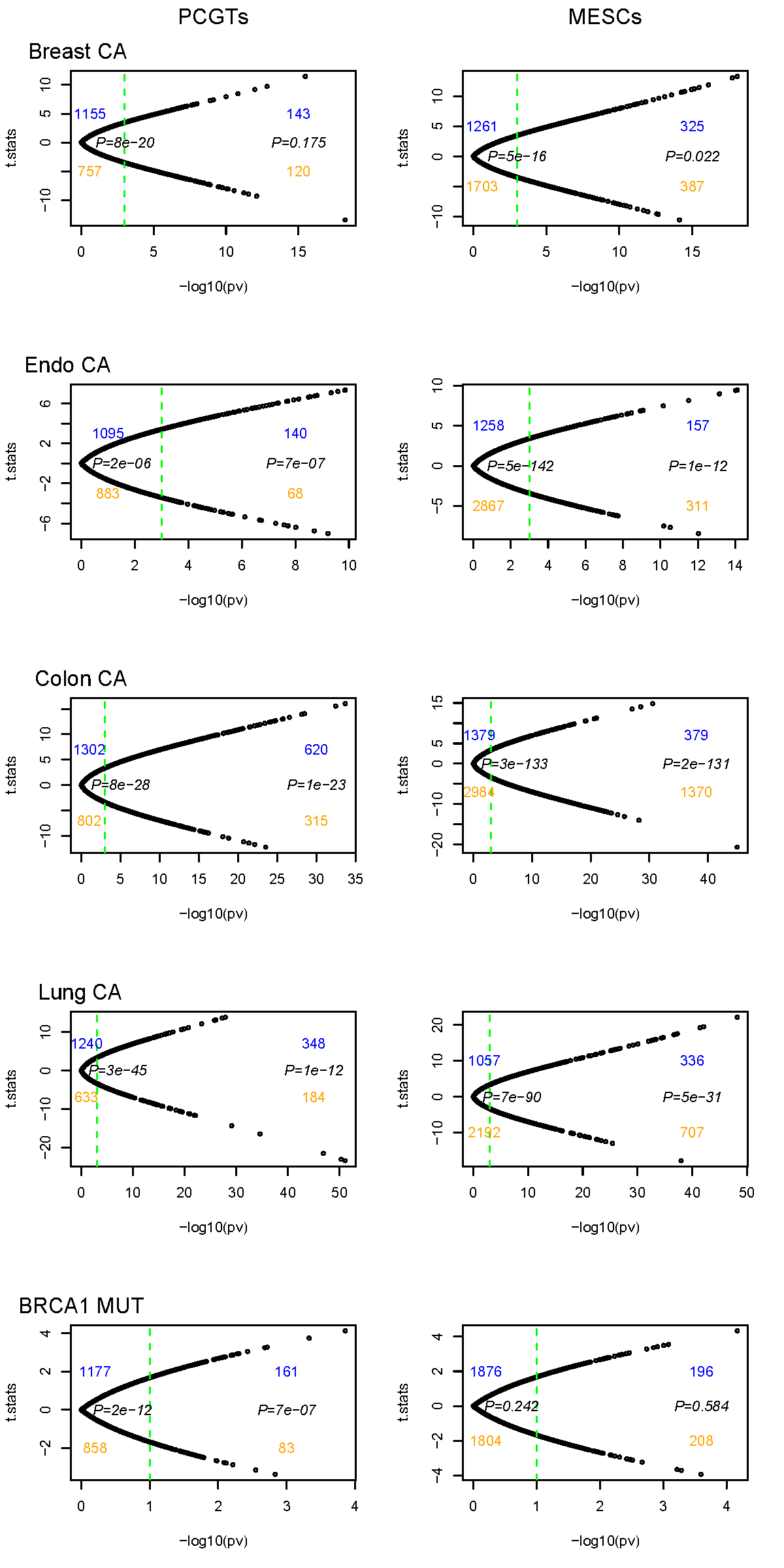

Supplement: Figure S7 — Statistical output from linear regression models estimating the association of the nonPMD PCGT and nonPMD MESC CpGs to the outcomes in five cohorts. Scatterplots of five cohorts, similar to Figure S6, based on the nonPMD PCGT (left column) and nonPMD MESC (right column) CpGs. (TIF) [file pgen.1002517.s007.tif]

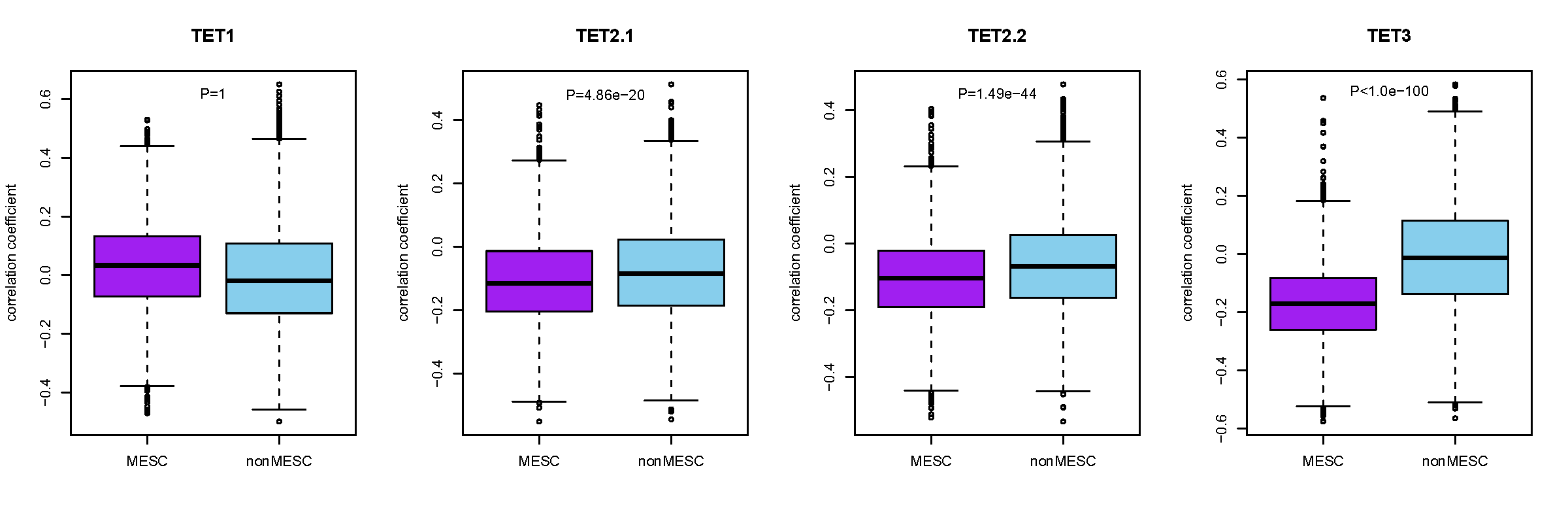

Supplement: Figure S8 — Magnitude of the anti-correlation between hypomethylated cervical cancer CpGs and TET mRNA. Hypomethylated MESCs are significantly higher anti-correlated with TET2 and TET3 mRNA expression levels than the hypomethylated nonMESCs in the cervical cancer samples. P-values are obtained from the Wilcoxon one-sided test. (TIF) [file pgen.1002517.s008.tif]

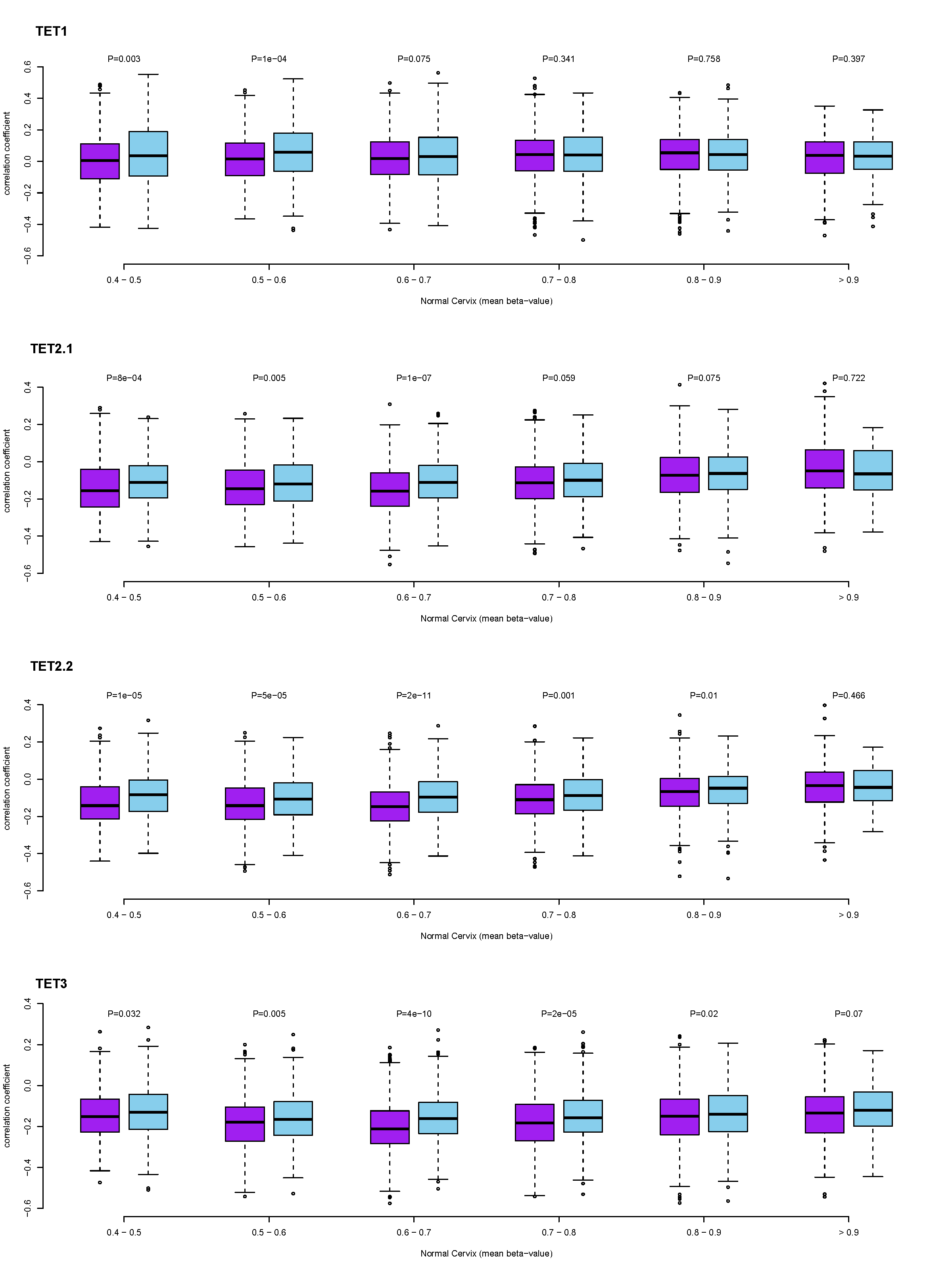

Supplement: Figure S9 — Magnitude of the anti-correlation between hypomethylated cervical cancer CpGs (grouped) and TET mRNA. Higher anti-correlated signature with TET2 and TET3 mRNA expression levels among hypomethylated MESCs than hypomethylated nonMESCs in the cervical cancer samples independent from the chosen baselines of the methylated and hemimethylated CpGs (mean β-value in normals >0.4). P-values are obtained from the Wilcoxon one-sided test. (TIF) [file pgen.1002517.s009.tif]

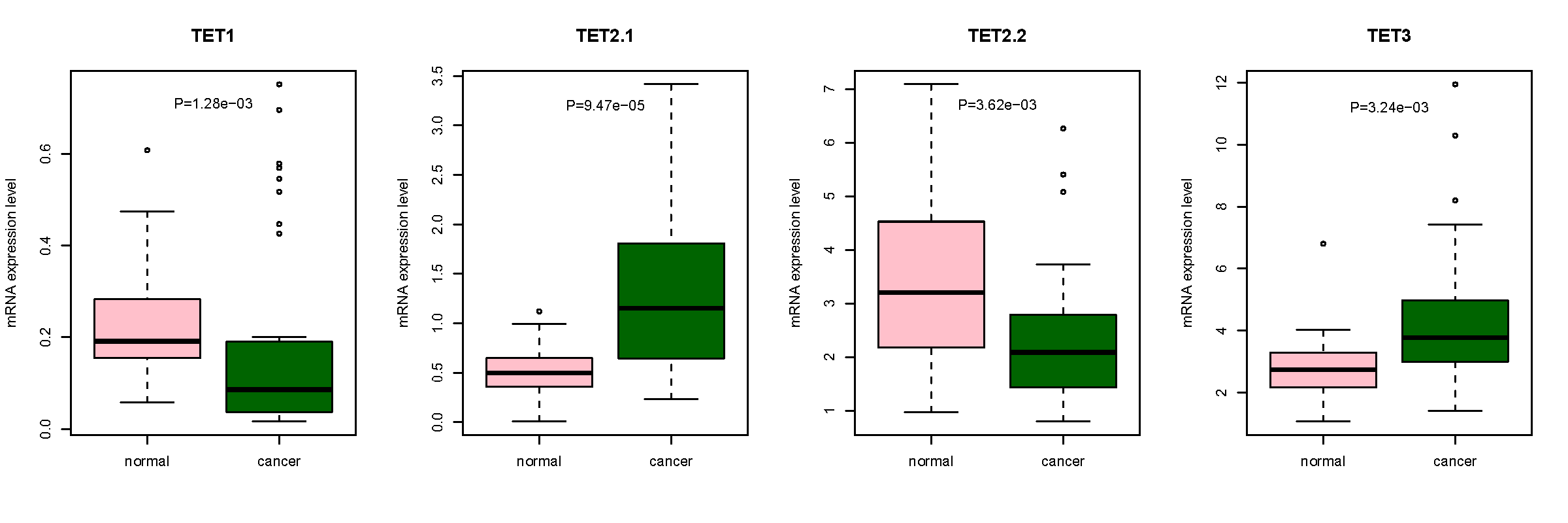

Supplement: Figure S10 — TET mRNA expression level comparison between the normal cervix and cervical cancers. Boxplots of TET1, TET2.1, TET2.2 and TET3 mRNA expression levels of the normal cervix and cervical cancers. P-values are obtained from the Wilcoxon two-sided test. (TIF) [file pgen.1002517.s010.tif]
