# Supplementary material for: The Dynamics and Prognostic Potential of DNA Methylation Changes at Stem Cell Gene Loci in Women's Cancer
Source: PLoS Genet. 2012 Feb 9;8(2):e1002517. doi: 10.1371/journal.pgen.1002517 (PMC3276553; doi:10.1371/journal.pgen.1002517)
Supplement: Text S1 — A detailed description of the definitions, study population, materials, and primers used in this study is provided. (DOC) [file pgen.1002517.s018.doc]

**TEXT S1**

- **Supporting Information for**

**“The Dynamics and Prognostic Potential of DNA Methylation Changes at Stem Cell Gene Loci in Women’s Cancer”**

by

Joanna Zhuang, Allison Jones, Shih-Han Lee, Esther Ng, Heidi Fiegl, Michal Zikan, David Cibula, Alexandra Sargent, Helga B. Salvesen, Usha Menon, Ian J Jacobs, Henry C. Kitchener, Andrew E. Teschendorff, Martin Widschwendter

- **Materials and Methods**

**Definitions**

**Definition of PMD**

***P***artially ***M***ethylated ***D***omains (PMDs) were originally defined as contiguous regions with an average methylation level less than 70% (mean length = 153 kb), which comprised a large proportion of every autosome, and over 80% of the IMR90 X chromosome . Over 40% of the IMR90 fibroblast genome falls within PMDs, and in IMR90 cells the transcript abundance associated with genes located within PMDs was lower than the average for all other genes . Here we used a definition of PMDs adopted from Berman et al. : they defined **IMR**90 fibroblast ***P***artially ***M***ethylated ***D***omains (IMR-PMDs) and colon tumour PMDs (colon-PMDs) as contiguous regions (longer than 100kb) of the IMR90 fetal lung fibroblasts genome and colon tumour genome respectively, with an average methylation level of 20-60%, collapsing these into non-overlapping domains .

- **Definition of MESC**
- A recent study used bisulfite sequencing to map, at single-base-resolution, DNA methylation throughout the majority of the human genome in both embryonic stem cells and fibroblasts . For each CpG site, the number of C and T reads covering each methyl cytosine on both forward and reverse strands were provided . The multiple reads covering each methyl cytosine can be used as readout of the fraction of sequences within the sample that are methylated at that particular site (i.e. C reads / C+T reads) , and hence, referred as the methylation level of the site. In this study, ***M***ethylated in human ***E***mbryonic ***S***tem ***C***ells (MESC) CpGs are the CpG sites that were covered by at least 5 reads on both forward and reverse strands (i.e. the total number of C and T reads on both strands >=5) and the overall mean methylation levels (i.e. the average methylation level of both the forward and reverse strands) is greater than 80%. MESC CpGs were then mapped to those present on the Illumina 27k array. Functional annotation (gene assignment) of the MESC CpGs present on the array was obtained from Illumina and Bioconductor annotation packages.
- **Definition of PCGTs**
- ***P***oly***C***omb ***G***roup ***T***arget genes (PCGTs) were defined as CpGs that are occupied by SUZ12 and/or EeD and/or are trimethylated at Lysine 27 on histone H3 in human embryonic stem cells (annotation file kindly provided by Benjamin P. Berman and Peter W. Laird).
- **Study Population**

Ethical approval was available for all samples in this study.

- **‘Before Dysplasia’ samples**
- The liquid based cytology (LBC) samples were collected from women (aged 19-55 years) as part of the ARTISTIC (A Randomized Trial In Screening To Improve Cytology) trial . These women were undergoing routine screening as part of the English National Health Service Cervical Screening Programme in Greater Manchester and were randomly assigned (between July 2001 and September 2003) to either combined LBC and human papillomavirus (HPV) testing in which the results were revealed and acted on, or to combined LBC and HPV testing where the HPV result was concealed from the patient and investigator. All women underwent two screening rounds with an interval of 3 years during which 75 women (mean age 27.4) developed Cervical Intraepithelial Neoplasia grade 2 or 3 (CIN2/3) whereas 77 (mean age 27.7) remained normal. Of the 75 samples that developed CIN2/3, 44 were HPV+ and 31 were HPV-, while of the 77 samples that remained normal, 48 were HPV+ and 29 HPV- (OR=0.86, P=0.74). Thus, samples were matched for age and HPV status. The methylation data have been deposited on Gene Expression Omnibus (GEO) under accession number GSE30760.
- **‘Dysplasia’ samples**
- 30 LBC samples (19 HPV- and 11 HPV+) from women (mean age 31.6) with normal cytology and 18 LBC samples (all HPV+) from women (mean age 31.3) with CIN2/3 . The methylation data is available from GEO: DataSet GSE20080.
- **‘Invasive Cancer’ samples**
- 63 cervical tissue samples with 48 cervical cancers (all HPV+) and 15 normal cervical tissue. The normal cervical tissue samples were from women (mean age 55.4 years) who underwent a hysterectomy for uterine ﬁbroids. Among the cancers, 26 were at stage 1 and 22 at stage 2/3/4; 7, 28 and 11 were grade 1, 2 and 3 respectively (grade information in two cancers is missing); and 36 were squamous cell cancers with the rest either adenocarcinoma or adenosquamous cervical cancers. The 48 cervical cancer specimens were from women (mean age 56.8 years) who were treated at the Innsbruck Medical University between 1990 and 2006. The methylation data are deposited in GEO: DataSet GSE30760.
- **Breast CA samples**
- 60 breast tissue samples with 37 breast cancers and 23 non-neoplastic breast tissues. The breast cancer tissue samples were from women (mean age 54.0) who were diagnosed with Estrogen-Receptor (ER) negative breast cancer. Among the cancers, 11 were at stage 1 and 26 at stage 2/3/4; 5, 19, 13 were grade 1, 2, and 3 respectively; and 29 were invasive ductal carcinoma breast cancers. The 23 non-neoplastic samples are from healthy woman (mean age 47.6). The methylation data has been published to GEO: DataSet GSE32393.
- **Breast CA (JHU) samples**
- 118 breast tissue samples with 103 primary invasive breast cancers (mean age 54) and 15 normal breast tissues (mean age 53). The breast cancer tissue samples were from women with Stage 1-3 disease prior to treatment from Surgical Pathology at Johns Hopkins Hospital (Baltimore, Maryland), among which 44 were diagnosed with Estrogen-Receptor (ER) positive and 38 with ER negative breast cancers (ER status of the remaining cancers is unavailable). 15 of the normal samples are enzymatic digestion of reduction mammoplasty specimens and 6 are normal ducts from breast tissue > 2 cm away from the tumour. The methylation data was obtained from GEO DataSet GSE31979.
- **Endo CA samples**
- 87 endometrium tissue samples with 64 endometrial cancers and 23 normal endometrial samples were collected in Bergen (Norway) and Innsbruck (Austria) respectively. The cancer tissue samples are from women (mean age 65.2) who were diagnosed with primary endometrial cancer. Among the cancers, 29,19, and 16 were at stage of 1A/B, 1C, and 2/3/4 (1988 International Federation of Gynecology and Obstetrics (FIGO) staging criteria) respectively, 19, 26, and 18 were grade 1, 2, and 3 respectively; and 56 were endometrioid and 8 non-endometrioid cancers. The 23 normal samples are from healthy women (mean age 43.1).

**Endo Meta CA samples**

17 endometrium tissue samples from metastatic endometrial cancers collected in Bergen (Norway) and Innsbruck (Austria) respectively. The samples are from women with mean age 67.1, among which 5, 3, and 9 were at stage of 1A/B, 1C, and 2/3/4 (1988 International Federation of Gynecology and Obstetrics (FIGO) staging criteria) respectively, 2 were at grade 2 and 15 grade 3; and 6 were endometrioid and 11 are non-endometrioid cancers. The methylation data has been published to GEO: DataSet GSE33422.

- **Ovarian CA Samples**
- 177 ovarian cancer tissue specimens from pre-and post-menopausal women (mean age 61.7). Among them, 7, 103, and 61 were grade 1, 2, and 3 (grade information of six cancers is not available) and 37, 11, 106, and 22 were at stage I, II, III, and IV (stage information of one cancer is unknot available) respectively . The distribution in terms of histology was: 76 serous, 57 mucinous, 24 endometrioid, 9 clear cell and 11 were classified as other histology.
- **Colon CA samples**
- 125 colorectal tumours (from 65 females and 60 males aged between 33 and 90) and 29 histologically normal adjacent colonic tissues from colorectal cancer patients and the methylation data were obtained from GEO DataSet GSE25062.
- **Lung CA Samples**
- 127 lung adenocarcinoma tumours and 24 matched normal lung tissues from The Cancer Genome Atlas (http://cancergenome.nih.gov/) Analysis of DNA Methylation for lung adenocarcinoma Using Illumina Infinium Human DNA Methylation 27 platform (HumanMethylation27).

**Lung CA Fibroblasts Samples**

5 non-small cell lung carcinoma fibroblasts (3 males and 2 females aged between 58 and 77; 2, 2 and 1 are at the stage of I, II, and III,) and matched normal lung fibroblasts . The methylation data was obtained from GEO DataSet GSE22874.

**TCGA ovarian CA Samples**

378 ovarian cancer tumour samples from woman with mean age 59.6, among which 19, 292, and 60 were at stage 2, 3, and 4 respectively (stage information in seven cancers is not available); and 2, 37, 325 and 1 were at grade 1,2,3, and 4 respectively (grade information in thirteen cancers is not available). The methylation data are obtained from The Cancer Genome Atlas (TCGA) Ovarian serous cystadenocarcinoma data set .

- ***BRCA1* MUT**

White Blood Cell (WBC) samples from *BRCA1* mutation carriers and *BRCA1* wild type controls. Whole blood samples were drawn from 30 *BRCA1* mutation carriers (15 breast cancers and 15 healthy with mean age 57.2) and 30 females without a *BRCA1* mutation (15 breast cancers and 15 healthy with mean age 57.1). All samples were collected between 2001 and 2008. The samples were drawn from women attending the General Faculty Hospital in Prague and the complete coding sequence, intron-exon junctions and large rearrangements for *BRCA1* and *BRCA2* genes were tested. All women gave their written informed consent and the use of the samples was approved by the local ethical committee. The methylation data has been published to GEO: DataSet GSE32396.

- **TET Primer Information**

The TET1 primers and probe were placed at the junction between exon 2 and 3 (amplicon location: NM_030625; 2384-2473). Forward: 5’-CTG AAA AAG AAA CCA TCT GTT GTT GT-3’; Reverse: 5’-CTT TAA AAC TTT GGG CTT CTT TTC C-3’; TaqMan Probe: 5’ FAM-TGG AGG TTA TAA AGG AAA ACA AGA GGC CCC-3’BHQ1.

The TET2.1 probe and primers were: (amplicon location: NM_001127208; 3893-3988) Forward: 5’-GTA GAG CAA ATT ATT GAA AAA GAT GAA GGT-3’; Reverse: 5’-CCT TTC TTC CAT GAT TTC TCT AAT AGC-3’; TaqMan Probe: 5’ FAM-CTT TTT ATA CCC ATC TAG GAG CAG GTC CTA ATG TGG-3’BHQ1.

The TET2.2 probe and primers were: (amplicon location: NM_001127208; 242-306) Forward: 5’-AGG AGA CCC GAC TGC AAC TG-3’; Reverse: 5’-TGT TTG CCA GCC TCG TTC T-3’; TaqMan Probe: 5’ FAM-TGG ATT GCT GCA AGG CTG AGG-3’BHQ1.

The TET3 primers and probe were placed at the junction between exon 3 and 4 (amplicon location: NM_144993; 2253-2310). Forward: 5’-CGG TTG CCC CAT TGC A-3’; Reverse: 5’-GCA CCA GGC AGA GTA GCT TCT C-3’; TaqMan Probe: 5’ FAM-ATC CGC AGG CAC ACG CTG GA-3’BHQ1.

**References**

- 1. Lister R PM, Dowen RH, Hawkins RD, Hon G, Tonti-Filippini J, Nery JR, Lee L, Ye Z, Ngo QM, (2009) Human DNA methylomes at base resolution show widespread epigenomic differences. Nature 462: 315-322.
- 2. Berman PB, Weisenberger DJ, Aman JF, Hinoue T, Ramjan Z, et al. (2011) Regions of focal DNA hypermethylation and long range hypomethylation in colorectal cancer coincide with nuclear lamina-associated domains. Nature Genetics.
- 3. Cokus SJ, Feng S, Zhang X, Chen Z, Merriman B, et al. (2008) Shotgun bisulphite sequencing of the Arabidopsis genome reveals DNA methylation patterning. Nature 452: 215-219.
- 4. Kitchener HC, Almonte M, Gilham C, Dowie R, Stoykova B, et al. (2009) ARTISTIC: a randomised trial of human papillomavirus (HPV) testing in primary cervical screening. Health Technol Assess 13: 1-150, iii-iv.
- 5. Teschendorff AE, Menon U, Gentry-Maharaj A, Ramus SJ, Weisenberger DJ, et al. (2010) Age-dependent DNA methylation of genes that are suppressed in stem cells is a hallmark of cancer. Genome Res 20: 440-446.
- 6. Salvesen HB, Carter SL, Mannelqvist M, Dutt A, Getz G, et al. (2009) Integrated genomic profiling of endometrial carcinoma associates aggressive tumors with indicators of PI3 kinase activation. Proc Natl Acad Sci U S A 106: 4834-4839.
- 7. Hinoue T, Weisenberger DJ, Lange CP, Shen H, Byun HM, et al. (2011) Genome-scale analysis of aberrant DNA methylation in colorectal cancer. Genome Res.
- 8. Navab R, Strumpf D, Bandarchi B, Zhu CQ, Pintilie M, et al. (2011) Prognostic gene-expression signature of carcinoma-associated fibroblasts in non-small cell lung cancer. Proc Natl Acad Sci U S A 108: 7160-7165.
- 9. Network CGAR (2011) Integrated genomic analyses of ovarian carcinoma. Nature 474: 609-615.
